# A Practical Approach to Bicyclic Carbamoyl Pyridones with Application to the Synthesis of HIV-1 Integrase Strand Transfer Inhibitors

**DOI:** 10.3390/molecules28031428

**Published:** 2023-02-02

**Authors:** Pankaj S. Mahajan, Steven J. Smith, Stephen H. Hughes, Xuezhi Zhao, Terrence R. Burke

**Affiliations:** 1 Chemical Biology Laboratory, Center for Cancer Research, National Cancer Institute, National Institutes of Health, Frederick, MD 21702, USA; 2 HIV Dynamics and Replication Program, Center for Cancer Research, National Cancer Institute, National Institutes of Health, Frederick, MD 21702, USA

**Keywords:** HIV-1, integrase strand transfer inhibitor (INSTI), one-pot synthesis, catalytic hydrogenation, bicyclic carbamoyl pyridones, metal chelation

## Abstract

An efficient one-pot synthetic method has been developed for the preparation of bicyclic carbamoyl pyridones from the known common intermediate methyl 5-((2,4-difluorobenzyl)carbamoyl)-1-(2,2-dimethoxyethyl)-3-methoxy-4-oxo-1,4-dihydropyridine-2-carboxylate (**8**). The scalable protocol is facile and employs readily available reagents, needing only a single purification as the final step. The utility of the approach was demonstrated by preparing a library of HIV-1 integrase strand transfer inhibitors (INSTIs) that differ by the presence or absence of a double bond in the B-ring of the bicyclic carbamoyl pyridines **6** and **7**. Several of the analogs show good antiviral potencies in single-round HIV-1 replication antiviral assays and show no cytotoxicity in cell culture assays. In general, the compounds with a B-ring double bond have higher antiviral potencies than their saturated congeners. Our methodology should be applicable to the synthesis of a range of new metal-chelating analogs.

## 1. Introduction

Bis-cationic magnesium (Mg^2+^) is the most abundant divalent cation in cells [1]. The hydration shell and ligand geometries of Mg^2+^ contribute to its role in polynucleotide folding and its frequent use by DNA and RNA polymerases and nucleases [2,3,4,5]. The critical roles played by divalent metal chelation in essential viral enzymes have made metal-chelating pharmacophores important components of a range of antiviral agents [6,7,8]. For example, HIV-1 replication involves the conversion of the single-stranded viral RNA genome into linear dsDNA by the polymerase and ribonuclease H (RNase H) activities of reverse transcriptase (RT), and subsequent incorporation of the viral DNA into the host genome through the catalysis of viral integrase (IN). Two Mg^2+^ cations serve as cognate catalytic cofactors for these enzymes [9,10,11]. Metal-chelating moieties are central components of inhibitors directed against both RNase H [12,13] and integrase [14,15,16,17,18,19,20,21]. Integrase strand transfer inhibitors (INSTIs) are of particular interest, with recently approved INSTIs representing some of most effective anti-AIDS drugs [22].

**Figure 1:**
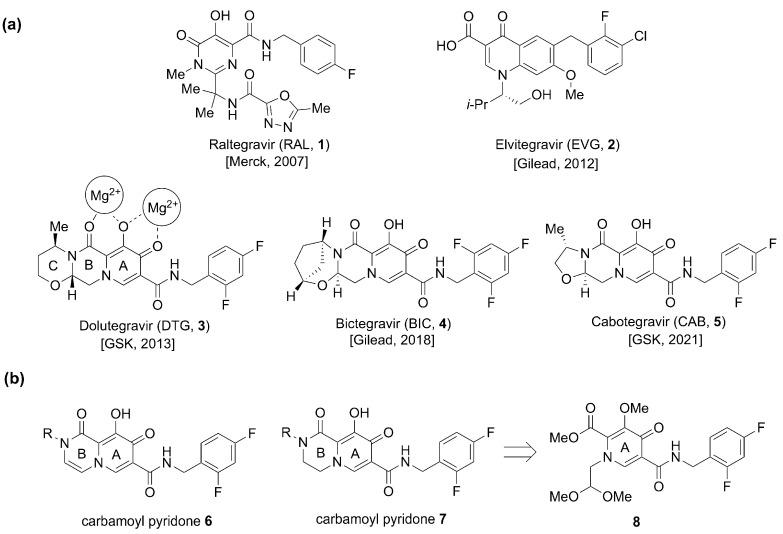
(**a**) FDA-approved INSTIs **1**–**5** and mode of metal chelation **3**. (**b**) Structures of bivalent metal-chelating carbamoyl pyridones **6** and **7** and the monocyclic synthetic precursor **8**.

Evolution of the metal-chelating motifs of INSTIs has played a key role in the improvements in the therapeutic efficacy of this class of drugs [14,20]. Of the five currently FDA-approved INSTIs (the first-generation INSTIs Raltegravir (RAL, **1**, approved in 2007) and Elvitegravir (EVG, **2**, approved in 2012), and second generation INSTIs Dolutegravir (DTG, **3**, approved in 2013), Bictegravir (BIC, **4**, approved in 2018), and Cabotegravir (CAB, **5**, approved in 2021)) the three second-generation drugs share a common metal-chelating platform based on carbamoyl pyridone motifs (9-hydroxy-2*H*-pyrido [1,2-*a*]pyrazine-1,8-dione **6** and the saturated congener 9-hydroxy-3,4-dihydro-2*H*-pyrido [1,2-*a*]pyrazine-1,8-dione (**7**, Figure 1) [3,4,21,23]. These three INSTIs have additional rings appended onto their carbamoyl pyridone cores. The focus of our current work is to improve the synthesis of metal-chelating carbamoyl pyridones from the monocyclic precursor **8** and to use this protocol for the synthesis of analogs bearing a variety of *N*-2-alkyl substituents (R, Figure 1). This work has led to the identification of potentially significant structure activity relationship (SAR) correlations.

## 2. Results and Discussion

### 2.1. Improved Synthetic Protocol to Prepare 2-Methyl Carbamoyl Pyridones ***6*** and ***7***

**Scheme 1:**
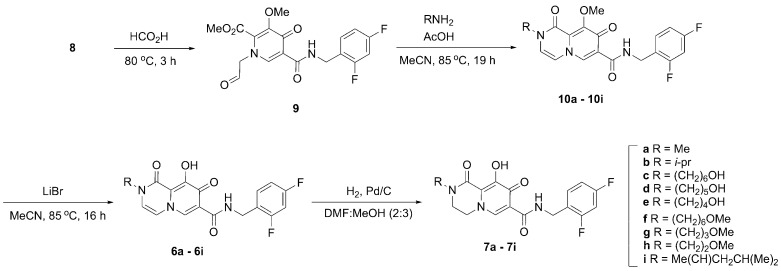
Synthetic approach to bicyclic carbamoyl pyridone derivatives **6** and **7**.

Synthesis of the bicyclic carbamoyl pyridone metal chelators of type **6** and **7** has previously involved multistep reaction sequences that entail purification after each step [24,25]. In some protocols the bicyclic pyridones have been obtained from aldehyde-containing monocyclic precursors of type **9** (Scheme 1) under microwave conditions [24]. Such harsh conditions may limit gram-scale synthesis. A more practical diversity-oriented synthesis from a common intermediate could potentially overcome these issues and generate a library of bicyclic pyridones through intermediate **10**. In this regard, we designed a synthetic protocol from the common precursor acetal **8** that produces bicyclic carbamoyl pyridones of type **6** and **7** in fewer steps without reliance on special reaction conditions. 

We began our synthesis with the common intermediate **8**, which can be synthesized on gram-scale [26] or obtained commercially. In our initial work, we heated **8** in formic acid at 80 °C to effect conversion to the aldehyde **9** (Scheme 1) [26,27,28]. Following evaporation of the solvent and drying under high vacuum, we obtained crude product **9** that was used directly for the next step. Methyl amine hydrochloride was added to aldehyde **9** followed by acetic acid and the reaction mixture was stirred at reflux. This afforded the bicyclic methyl-protected carbamoyl pyridone **10a** in moderate yields (44% yield over two steps). We examined the reaction under microwave conditions (140 °C, 1 h) as previously reported [24]. However, those conditions resulted in lower yields (11%). The pyridone **10a** was subsequently demethylated using lithium bromide to yield the final carbamoyl pyridone **6a** in 60% yield after Prep-HPLC purification. In this way, we were able to convert **8** to **6a** in 26% combined yield in three steps using two purifications (Scheme 1). 

We envisioned a more straightforward one-pot synthesis of **6a** directly from the common intermediate **8** that would not require intermediate purification. Accordingly, the reaction sequence described above was repeated in a one-pot synthesis. The methyl-protected enol **10a** was not isolated directly. Rather, following the conversion of **10a** to **6a**, solvent and acetic acid were removed by rotary evaporation and the resulting residue was placed under high vacuum (30 min). Conversion of **8** to enol **6a** was achieved in one-pot fashion in 31% yield over three steps following HPLC purification (Scheme 1). 

### 2.2. Synthesis of Libraries of 2-Alkyl Bicyclic Carbamoyl Pyridones

In clinically approved second-generation INSTIs, a heterocyclic functionality positioned to the “left” of the core carbamoyl bicyclic pyridone-containing metal-chelating platform can have a significant impact on the ability of the compounds to potently inhibit drug-resistant IN variants (Figure 1). Although such INSTIs have been developed empirically, a rationale for the potential roles played by this functionality is beginning to emerge from high resolution structures of INSTIs bound to intasomes, where it is found that this region of the INSTI contacts the β4-α2 loop of IN and helps the compounds retain better potency against drug-resistant integrase mutants [20,22,29]. In the current work, we used the optimized carbamoyl pyridone synthetic protocol to derive libraries of bicyclic analogs having varied *N*-alkyl functionality that would project from this “left” region of the INSTI. We prepared nine compounds with a double bond in the B-ring (**6a**–**6i**, Scheme 1). Catalytic hydrogenation of the double bond in the B-ring afforded a parallel set of nine reduced molecules (**7a**–**7i**, Scheme 1).

### 2.3. Determination of HIV-1 Antiviral Potencies

The bicyclic metal chelating carbamoyl pyridone moiety is common to second-generation tricyclic INSTIs (**3**, **4**, and **5**, Figure 1). Analogs of the current study are simplified analogs that lack a third ring. While our compounds contain a 2,4-difluorobenzyl amide group, structurally similar bicyclic carbamoyl pyridones possessing a 4-fluorobenzyl amide group have been previously reported [24]. The halogen substitution pattern of the benzylamide moiety can affect inhibitory potencies of INSTIs [30]. We determined the antiviral potencies of our compounds in single round HIV-1 replication assays using viral constructs with wild-type IN [31]. Cytotoxicities were determined as previously described [31]. The FDA-approved INSTIs **3**, **4**, and **5** showed similar antiviral EC_50_ values of approximately 2 nM (Table 1). In general, the potencies of bicyclic analogs **6** having an unsaturated bond at the C4 position were insensitive to differences in *N*-methyl substituents. Most compounds uniformly exhibited single digit nanomolar antiviral potencies, with the exception of **6d** and **6e**, which had antiviral EC_50_ values of approximately 11 nM and 17 nM, respectively. Members of the unsaturated series **6** were uniformly found to exhibit low cytotoxicities (CC_50_ > 250 nM), except for the *N*-methyl substituted analog **6a**, which showed slightly increased cytotoxicity (CC_50_ = 121 nM). In the case of isopropyl substitution (**6b** and **7b**) antiviral potencies were similar; however, cytotoxicities increased slightly after reduction of the double bond (CC_50_ > 250 nM and 167 nM, respectively). A slight loss of antiviral potency was observed with analogs having terminal hydroxy groups in the unsaturated series **6c**, **6d**, and **6e**, (antiviral EC_50_ values of 4.8 nM, 10.8 nM, and 16.6 nM, respectively) with an even greater loss of potency for their corresponding saturated analogs **7c**, **7d**, and **7e** (EC_50_ values of 31.7 nM, 54.5 nM, and 75.7 nM, respectively). The unsaturated series compound **6f**, which is a methyl-ether congener of primary alcohol **6c**, does not have improved antiviral potency relative to the unsaturated version **7f** (EC_50_ = 4.6 nM). However, conversion of the unsaturated analog **6c** to yield compound **7c** resulted in a six-fold decrease in potency relative to **6c**. Unsaturated analogs having terminal methoxy-containing *N*-substituents (**6g** and **6h**) showed antiviral potencies that were similar to the second-generation tricyclic INSTIs **3**, **4,** and **5** (EC_50_ ≈ 2 nM for both series). Analogs **6g** and **6h** represent simplified analogs of DTG (**3**) and CAB (**5**), respectively, in which the third ring (C-ring) is opened and having doble bond in B-ring, but lacking α-methyl stereochemistry.

To further evaluate the antiviral activities of select compounds, we determined their EC_50_ values against the IN mutant S230N, which has been selected in vitro against an early Merck INSTI L810,830, a prototype for naphthyridine analogs [32]. Additionally, in our single round infection assays, we have shown that the S230N mutant displays a modest drop in susceptibility to DTG (7.9± 1.3 nM), which is atypical for an IN single mutant [33]. Compounds **6d** (15.4 ± 0.3 nM), **6e** (30.7 ± 1.4 nM), **7c** (38.0 ± 3.8 nM), and **7e** (100.8 ± 17.5 nM) all retained most of their potency against the IN mutant S230N, while compounds **6c** (9.3 ± 1.9 nM) and **7d** (97.0 ± 16.9 nM), displayed minor losses in potency against this IN mutant (Table 2).

### 2.4. Potential Role of Unsaturation in the B-Ring

**Figure 2:**
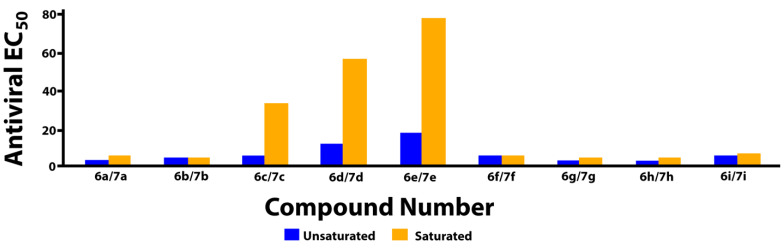
Comparison of antiviral potencies for saturated versus unsaturated analogs.

**Figure 3:**
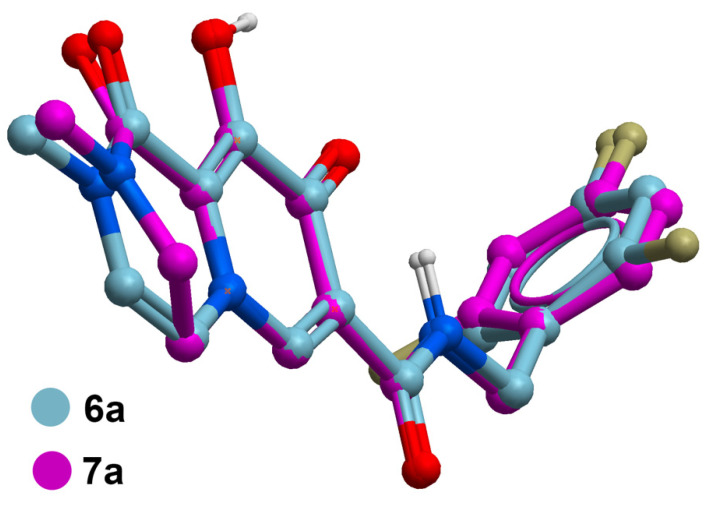
Superposition of in silico generated minimized structures of unsaturated **6a** with the corresponding saturated analog **7a** showing buckling of the respective B-rings that leads to a slight out-of-plane tilting of the B-ring carboxamide carbonyl. Structure generation and minimization was performed with MolSoft ICM software using standard parameters [35].

The data in Table 1 reveal that the bicyclic carbamoyl pyridones having a double bond in their B-ring exhibit higher antiviral potencies than their saturated counterparts. A graphical comparison of antiviral potencies for unsaturated versus saturated congeners is provided (Figure 2). Unsaturation in the B-ring may contribute to improved antiviral potency for at least two reasons. First, as shown by superimposing in silico generated minimized structures of **6a** and **7a** (Figure 3), the double bond in the bicyclic system results in greater planarity as compared to the more buckled ring system of the saturated congeners**.** This results in a slight out-of-plane alignment of the metal-chelating B-ring amide carbonyl oxygen. Maintaining planarity of chelating heteroatoms may potentially contribute to more facile Mg^2+^ chelation. Second, by forming an electron-rich aromatic system, unsaturation in the B-ring may also contribute to improved potency by enhancing stacking interactions of the bicyclic carbamoyl pyridone ring system with the terminal adenosine nucleobase of the viral DNA. Stacking of the adenosine with the INSTI has been suggested to favor binding [34].

## 3. Conclusions

We present one-pot synthetic methodology that yields a common bicyclic carbamoyl platform starting from the acetal intermediate **8**. This represents an improvement in previous multistep approaches, which had required special reaction conditions and purification at each step. The new molecules synthesized in our current manuscript represent simplified analogs of the FDA-approved second-generation tricyclic INSTIs. Single digit nanomolar antiviral potencies were observed for several compounds, with analogs **6g** and **6h** having antiviral potencies similar to the FDA-approved second-generation INSTIs. The antiviral data suggests that there is the potential importance of a double bond in the B-ring. Improved potency of the unsaturated compounds may derive from induced planarity in the bicyclic carbamoyl pyridone rings as well as improved stacking interactions with the viral DNA terminal adenosine nucleobase. Our work opens the possibility of delivering potential bicyclic and tricyclic carbamoyl pyridone-containing INSTIs (including FDA-approved second-generation INSTIs) from appropriate amines/amino alcohols in a one-pot operation with a single purification. The synthesis and bio-evaluation of analogs using the disclosed protocol is currently underway in our laboratory.

## 4. Experimental Section

### 4.1. General Synthesis

Proton (^1^H) and carbon (^13^C) NMR spectra (see the Supplementary Materials) were recorded on a Varian 400 MHz spectrometer and are reported in ppm relative to TMS and referenced to the solvent in which the spectra were collected. Solvent was removed by rotary evaporation under reduced pressure, and anhydrous solvents were obtained commercially and used without further drying. Purification by silica gel chromatography was performed using Combi flash with EtOAc−hexanes or MeOH in DCM solvent systems; otherwise noted. Preparative high-pressure liquid chromatography (Prep-HPLC) was conducted using a Waters Prep LC4000 system having photodiode array detection and Phenomenex C18 columns (catalog no. 00G-4436-P0-AX, 250 mm × 21.2 mm 10 μm particle size, 110 Å pore) at a flow rate of 20 mL/min. Binary solvent systems consisting of A = 0.1% aqueous TFA and B = 0.1% TFA in acetonitrile were employed with linear gradients from 0–100% B. Products were obtained as amorphous solids following lyophilization. Electrospray ionization-mass spectrometric (ESI-MS) results were acquired with an Agilent LC/MSD system equipped with a multimode ion source. Purities of samples subjected to biological testing were assessed using this system and shown to be ≥95%. High resolution mass spectrometric (HRMS) readings were acquired by LC/MS-ESI using LTQ-Orbitrap-XL at 30K resolution. All the reactions were carried out under inert atmosphere using an argon balloon; otherwise noted.

### 4.2. Methyl 5-((2,4-Difluorobenzyl)carbamoyl)-1-(2,2-dimethoxyethyl)-3-methoxy-4-oxo-1,4-dihydropyridine-2-carboxylate (***8***)

Common intermediate **8** was synthesized according to literature procedures [26], mp 85–90 °C; ^1^H NMR (400 MHz, CDCl_3_) δ 10.38 (t, *J* = 5.3 Hz, 1 H), 8.41 (s, 1H), 7.42–7.33 (m, 1H), 6.87-6.75 (m, 2H), 4.63 (d, *J* = 5.3 Hz, 2 H), 4.50 (t, *J* = 4.8 Hz, 1H), 4.04 (d, *J* = 4.8 Hz, 2H), 3.99 (s, 3H), 3.96 (s, 3H), 3.39 (s, 6H); ^13^C NMR (101 MHz, CDCl_3_) δ 173.2, 164.1, 162.2 (dd, *J* = 248, 12 Hz, 1C), 162.2, 160.8 (dd, *J* = 248, 12 Hz, 1C), 149.4, 144.5, 134.9, 130.7 (dd, *J* = 9.6, 5.8 Hz, 1C), 121.5 (dd, *J* = 15.1, 3.6 Hz, 1C), 119.3, 111.1 (dd, *J* = 21.1, 3.7 Hz, 1C), 103.7 (t, *J* = 25.4, 1C), 102.7, 60.7, 56.8, 55.6, 53.4, 36.5 (d, *J* = 3.8, 1C); ESI-MS: 441 (M + H).

### 4.3. N-(2,4-Difluorobenzyl)-9-methoxy-2-methyl-1,8-dioxo-1,8-dihydro-2H-pyrido [1,2-a]pyrazine-7-carboxamide (***10a***)

To a 25 mL single-necked round bottom flask equipped with a magnetic stirring bar and a reflux condenser was added and acetal **8** (500 mg, 1.14 mmol, 1.0 equiv.) and formic acid (20 mL) and the resulting mixture was heated at 80 °C under an inert atmosphere using argon balloon for the next 3 h until the LCMS indicates complete consumption of the starting material to corresponding aldehyde **9**. The solvent formic acid was evaporated over rotavapor, and a high vacuum was applied to remove traces of formic acid. To the crude aldehyde **9**, was added dry acetonitrile (15 mL) followed by the addition of methylamine hydrochloride (230 mg, 3.4 mmol, 3.0 equiv.) and acetic acid (0.23 mL, 3.97 mmol, 3.5 equiv.), and the resulting reaction mixture was refluxed for next 16 h; the product **10a** was confirmed by LCMS. After completion of the reaction the solvent was removed, the residue was dissolved in DCM (50 mL), and washings (3 × 15 mL H_2_O) were given. The organic layer was separated and dried over sodium sulfate and concentrated. The crude product was then purified by Combi-Flash (MeOH in DCM; 1–10%) to afford the expected compound as a white solid (187 mg, 44% yield over two steps). ^1^H NMR (400 MHz, CDCl_3_) δ 10.55 (t, *J* = 5.9 Hz, 1H), 8.60 (s, 1H), 7.38 (td, *J* = 8.7, 6.5 Hz, 1H), 6.89–6.76 (m, 2H), 6.67 (d, *J* = 6.2 Hz, 1H), 6.42 (d, *J* = 6.2 Hz, 1H), 4.66 (d, *J* = 5.9 Hz, 2H), 4.07 (s, 3H), 3.42 (s, 3H). ^13^C NMR (101 MHz, CDCl_3_) δ 172.8, 163.6, 162.1 (dd, *J* = 248.2, 11.9, 1C), 161.1 (dd, *J* = 249.3, 12.0, 1C), 154.5, 153.5, 137.6, 130.6 (dd, *J* = 9.6, 5.7, 1C), 128.9, 121.2 (dd, *J* = 15.4, 4.0, 1C), 120.8, 119.9, 111.2 (dd, *J* = 21.2, 3.8, 1C), 110.7, 103.8 (t, *J* = 25.3, 1C), 61.1, 36.7 (d, *J* = 3.8, 1C), 36.1. ESI-MS m/z: 376.00 (M + H)^+^.

### 4.4. N-(2,4-Difluorobenzyl)-9-hydroxy-2-methyl-1,8-dioxo-1,8-dihydro-2H-pyrido [1,2-a]pyrazine-7-carboxamide (***6a***)

To a 25 mL single-necked round bottom flask equipped with reflux condenser and magnetic stirring bar was added compound **10a** (200 mg, 0.5 mmol, 1 equiv.) and lithium bromide (102 mg, 1.172 mmol, 2.2 equiv.) followed by addition of dry acetonitrile (10 mL) under argon atmosphere and the reaction was refluxed for the next 12–16 h. Once the reaction was complete as indicated by the LCMS, the reaction was cooled to room temperature and acetic acid (91 µL, 1.599 mmol, 3 equiv. in 3 mL H_2_O) was added to the reaction and stirred for the next 3 h at ambient temperature. Acetonitrile was removed over rotavapor and the resulting suspension of the final enone product **6a** in H_2_O was filtered through Whatman’s filter paper. The filtrate was again diluted with H_2_O (5 mL × 3) and the resulting solid product was collected by filtration. The product was then dissolved in DMSO and purified by Prep-HPLC using a binary mixture of 0.1% TFA in acetonitrile and 0.1% TFA in H_2_O as eluents to afford the product **6a** as a white solid (117 mg, 60% yield). ^1^H NMR (400 MHz, DMSO-*d*_6_) δ 12.09 (s, 1H), 10.59 (t, *J* = 5.9 Hz, 1H), 8.78 (s, 1H), 7.48 (d, *J* = 6.2 Hz, 1H), 7.46–7.38 (m, 1H), 7.29–7.20 (m, 1H), 7.10–7.02 (m, 1H), 6.92 (d, *J* = 6.2 Hz, 1H), 4.57 (d, *J* = 5.9 Hz, 2H), 3.32 (s, 3H). ^13^C NMR (101 MHz, DMSO-*d*_6_) δ 168.0, 163.4, 161.5 (dd, *J* = 245.2, 12.3 Hz, 1C), 160.3 (dd, *J* = 247.6, 12.6 Hz, 1C), 152.6, 134.9, 130.8 (dd, *J* = 10.0, 6.2 Hz, 1C), 122.2 (dd, *J* = 15.1, 3.5 Hz, 1C), 119.8, 117.8, 117.3, 112.8, 111.3 (dd, *J* = 20.9, 3.6 Hz, 1C), 103.8 (t, *J* = 25.7 Hz, 1C), 35.8 (d, *J* = 3.5 Hz, 1C), 34.8. HRMS-ESI (m/z) calcd for (C_17_H_13_F_2_N_3_O_4_ + H)^+^: 362.0947, found: 362.0955.

### 4.5. General Procedure I: One-Pot Synthesis of ***6a***–***6i***

To a 25 mL single-necked round bottom flask equipped with a magnetic stirring bar and a reflux condenser was added and acetal **8** (1 equiv.) and formic acid (20 mL for 1 g) and the resulting mixture were heated at 80 °C under an inert atmosphere using argon balloon for the next 3 h until the LCMS indicates complete consumption of the starting material to the corresponding aldehyde. The solvent formic acid was evaporated over rotavapor, and a high vacuum was applied to remove traces of formic acid. To the crude aldehyde **9**, was added dry acetonitrile (20 mL for 1 g) followed by the addition of amine (3 equiv.) and acetic acid (3.5 equiv.), and the resulting reaction mixture was refluxed for the next 16 h; the product **10** was confirmed by LCMS. The solvent was evaporated on rotavapor, and a high vacuum was applied to remove solvent traces. To the crude product **10**, was added lithium bromide (2.2 equiv.) followed by the addition of dry acetonitrile (20 mL for 1 g) under argon atmosphere, and the reaction was refluxed for the next 12–16 h. Once the reaction was complete as indicated by the LCMS, the reaction was cooled to room temperature and acetic acid (3 equiv. in 3–5 mL H_2_O) was added to the reaction and stirred for the next 3 h at ambient temperature. Acetonitrile was removed over rotavapor and the resulting suspension of the final enone product **6** in H_2_O was filtered through Whatman’s filter paper and the filtrate was again diluted with H_2_O (5 mL × 3) and the resulting solid product was collected by filtration. The product was then dissolved in DMSO and purified by Prep-HPLC.

### 4.6. N-(2,4-Difluorobenzyl)-9-hydroxy-2-methyl-1,8-dioxo-1,8-dihydro-2H-pyrido [1,2-a]pyrazine-7-carboxamide (***6a***)

White solid (267 mg, 31% yield from 1 g of **8**), mp 275–280 °C; ^1^H NMR (400 MHz, DMSO-*d*_6_) δ 12.09 (s, 1H), 10.59 (t, *J* = 5.9 Hz, 1H), 8.78 (s, 1H), 7.48 (d, *J* = 6.2 Hz, 1H), 7.46–7.38 (m, 1H), 7.29–7.20 (m, 1H), 7.10–7.02 (m, 1H), 6.92 (d, *J* = 6.2 Hz, 1H), 4.57 (d, *J* = 5.9 Hz, 2H), 3.32 (s, 3H). ^13^C NMR (101 MHz, DMSO-*d*_6_) δ 168.0, 163.4, 161.5 (dd, *J* = 245.2, 12.3 Hz, 1C), 160.3 (dd, *J* = 247.6, 12.6 Hz, 1C), 152.6, 134.9, 130.8 (dd, *J* = 10.0, 6.2 Hz, 1C), 122.2 (dd, *J* = 15.1, 3.5 Hz, 1C), 119.8, 117.8, 117.3, 112.8, 111.3 (dd, *J* = 20.9, 3.6 Hz, 1C), 103.8 (t, *J* = 25.7 Hz, 1C), 35.8 (d, *J* = 3.5 Hz, 1C), 34.8. HRMS-ESI (m/z) calcd for (C_17_H_13_F_2_N_3_O_4_ + H)^+^: 362.0947, found: 362.0955.

### 4.7. N-(2,4-Difluorobenzyl)-9-hydroxy-2-isopropyl-1,8-dioxo-1,8-dihydro-2H-pyrido [1,2-a]pyrazine-7-carboxamide (***6b***)

White solid, 29% yield, 130 mg from 500 mg of **8**, mp 180-182 °C; ^1^H NMR (400 MHz, DMSO-*d*_6_) δ 12.26 (s, 1H), 10.61 (t, *J* = 5.9 Hz, 1H), 8.80 (s, 1H), 7.56 (d, *J* = 6.4 Hz, 1H), 7.48–7.41 (m, 1H), 7.30–6.21 (m, 1H), 7.13–7.02 (m, 1H), 7.05 (d, *J* = 6.4 Hz, 1H), 4.88 (hept, *J* = 6.8 Hz, 1H), 4.57 (d, *J* = 5.9 Hz, 2H), 1.29 (d, *J* = 6.8 Hz, 6H). ^13^C NMR (101 MHz, DMSO-*d*_6_) δ 168.1, 163.4, 161.5 (dd, *J* = 245.5, 12.2 Hz, 1C), 160.3 (dd, *J* = 247.4, 12.3 Hz, 1C), 160.2, 158.4, 158.1, 153.1, 134.8, 130.9 (dd, *J* = 9.8, 6.0 Hz, 1C), 122.2 (dd, *J* = 15.2, 3.7 Hz, 1C), 117.7, 117.3, 114.3, 113.6, 111.4 (dd, *J* = 21.1, 3.6 Hz, 1C), 103.8 (t, *J* = 25.7 Hz, 1C), 45.8, 35.8 (d, *J* = 3.6 Hz, 1C), 19.9. HRMS-ESI (m/z) calcd for (C_19_H_17_F_2_N_3_O_4_ + H)^+^: 390.1260, found: 390.1267.

### 4.8. N-(2,4-Difluorobenzyl)-9-hydroxy-2-(6-hydroxyhexyl)-1,8-dioxo-1,8-dihydro-2H-pyrido [1,2-a]pyrazine-7-carboxamide (***6c***)

White solid, 28% yield, 290 mg from 1 g of **8**, mp 216-220 °C; ^1^H NMR (400 MHz, DMSO-*d*_6_) δ 12.14 (s, 1H), 10.59 (t, *J* = 5.8 Hz, 1H), 8.77 (s, 1H), 7.50 (d, *J* = 6.2 Hz, 1H), 7.46–7.39 (m, 1H), 7.29–7.22 (m, 1H), 7.07–7.11 (m, 1H), 6.96 (d, *J* = 6.2 Hz, 1H), 4.56 (d, *J* = 5.8 Hz, 2H), 4.33 (bs, 1H), 3.74 (t, *J* = 7.2 Hz, 2H), 3.38 (d, *J* = 6.4 Hz, 2H), 1.70–1.57 (m, 2H), 1.47–1.36 (m, 2H), 1.36–1.24 (m, 4H). ^13^C NMR (101 MHz, DMSO-*d*_6_) δ 168.1, 163.4, 161.5 (dd, *J* = 245.4, 12.1 Hz, 1C), 160.6, 160.2 (dd, *J* = 247.3, 12.4 Hz, 1C), 152.9, 134.9, 130.8 (dd, *J* = 19.9, 6.1 Hz, 1C), 122.2 (dd, *J* = 15.2, 3.7 Hz, 1C), 118.7, 117.8, 117.3, 113.1, 111.3 (dd, *J* = 21.1, 3.6 Hz, 1C), 103.8 (t, *J* = 25.8 Hz, 1C), 60.5, 47.0, 35.8 (d, *J* = 3.4 Hz, 1C), 32.3, 27.6, 25.8, 25.1. HRMS-ESI (m/z) calcd for (C_22_H_23_F_2_N_3_O_5_ + H)^+^: 448.1679, found: 448.1690.

### 4.9. N-(2,4-Difluorobenzyl)-9-hydroxy-2-(5-hydroxypentyl)-1,8-dioxo-1,8-dihydro-2H-pyrido [1,2-a]pyrazine-7-carboxamide (***6d***)

White solid, 15% yield, 103 mg from 700 mg of **8**, mp 203-205 °C; ^1^H NMR (400 MHz, DMSO-*d*_6_) δ 12.14 (s, 1H), 10.59 (t, *J* = 5.9 Hz, 1H), 8.78 (s, 1H), 7.50 (d, *J* = 6.2 Hz, 1H), 7.48–7.37 (m, 1H), 7.30–7.20 (m, 1H), 7.11–7.03 (m, 1H), 6.96 (d, *J* = 6.2 Hz, 1H), 4.57 (d, *J* = 5.9 Hz, 2H), 4.37 (bs, 1H), 3.74 (t, *J* = 7.2 Hz, 2H), 3.38 (t, *J* = 6.3 Hz, 2H), 1.65 (p, *J* = 7.5 Hz, 2H), 1.44 (dt, *J* = 8.6, 6.2 Hz, 2H), 1.32 (tt, *J* = 9.6, 5.7 Hz, 2H). ^13^C NMR (101 MHz, DMSO-*d*_6_) δ 168.1, 163.4, 161.5 (dd, *J* = 245.5, 12.3 Hz, 1C), 160.6, 160.2 (dd, *J* = 247.0, 12.3 Hz, 1C), 152.9, 134.9, 130.8 (dd, *J* = 9.8, 6.0 Hz, 1C), 122.2 (dd, *J* = 15.2, 3.6 Hz, 1C), 118.7, 117.8, 117.3, 113.1, 111.4 (dd, *J* = 21.1, 3.6 Hz, 1C), 103.8 (t, *J* = 25.7 Hz, 1C), 60.4, 47.0, 35.8 (d, *J* = 3.5 Hz, 1C), 32.0, 27.5, 22.5. HRMS-ESI (m/z) calcd for (C_21_H_21_F_2_N_3_O_5_ + H)^+^: 434.1522, found: 434.1531.

### 4.10. N-(2,4-Difluorobenzyl)-9-hydroxy-2-(4-hydroxybutyl)-1,8-dioxo-1,8-dihydro-2H-pyrido [1,2-a]pyrazine-7-carboxamide (***6e***)

White solid, 10% yield, 100 mg, from 1 g of **8**, mp 207-210 °C; ^1^H NMR (400 MHz, DMSO-*d*_6_) δ 12.14 (s, 1H), 10.59 (t, *J* = 5.9 Hz, 1H), 8.77 (s, 1H), 7.50 (d, *J* = 6.2 Hz, 1H), 7.45–7.38 (m, 1H), 7.26–7.19 (m, 1H), 7.10–7.03 (m, 1H), 6.96 (d, *J* = 6.2 Hz, 1H), 4.56 (d, *J* = 5.9 Hz, 2H), 4.46 (bs, 1H), 3.76 (t, *J* = 7.2 Hz, 2H), 3.44–3.39 (m, 2H), 1.68 (p, *J* = 7.5 Hz, 2H), 1.44 (dt, *J* = 13.1, 6.4 Hz, 2H). ^13^C NMR (101 MHz, DMSO-*d*_6_) δ 168.1, 163.4, 161.5 (dd, *J* = 245.4, 12.1 Hz, 1C), 160.6, 160.3 (dd, *J* = 247.3, 12.3 Hz, 1C), 160.20, 152.9, 134.9, 130.94, 130.8 (dd, *J* = 9.8, 6.1 Hz, 1C), 122.2 (dd, *J* = 15.2, 3.7 Hz, 1C), 118.7, 117.8, 117.3, 113.1, 111.4 (dd, *J* = 21.1, 3.6 Hz, 1C), 103.83 (t, *J* = 25.7 Hz, 1C), 60.2, 46.9, 35.9 (d, *J* = 3.5 Hz, 1C), 29.3, 24.5. HRMS-ESI (m/z) calcd for (C_20_H_19_F_2_N_3_O_5_ + H)^+^: 420.1366, found: 420.1370.

### 4.11. N-(2,4-Difluorobenzyl)-9-hydroxy-2-(6-methoxyhexyl)-1,8-dioxo-1,8-dihydro-2H-pyrido [1,2-a]pyrazine-7-carboxamide (***6f***)

White solid, 23% yield, 120 mg from 500 mg of **8**, mp 188-190 °C; ^1^H NMR (400 MHz, DMSO-*d*_6_) δ 12.14 (s, 1H), 10.59 (t, *J* = 5.9 Hz, 1H), 8.78 (s, 1H), 7.50 (d, *J* = 6.3 Hz, 1H), 7.47–7.38 (m, 1H), 7.30–7.21 (m, 1H), 7.11–7.02 (m, 1H), 6.96 (d, *J* = 6.3 Hz, 1H), 4.57 (d, *J* = 5.9 Hz, 2H), 3.77–3.69 (m, 2H), 3.29 (t, *J* = 6.5 Hz, 2H), 3.20 (s, 3H), 1.69–1.59 (m, 2H), 1.48 (td, *J* = 10.1, 8.5, 5.3 Hz, 2H), 1.31 (dd, *J* = 6.9, 3.5 Hz, 4H). ^13^C NMR (101 MHz, DMSO-*d*_6_) δ 168.1, 163.4, 161.5 (dd, *J* = 245.6, 12.2 Hz, 1C), 160.6, 160.3 (dd, *J* = 247.5, 12.4 Hz, 1C), 152.9, 134.9, 130.8 (dd, *J* = 9.9, 6.3 Hz, 1C), 122.2 (dd, *J* = 15.2, 3.6 Hz, 1C), 118.7, 117.8, 117.3, 113.1, 111.4 (dd, *J* = 21.0, 3.6 Hz, 1C), 103.8 (t, *J* = 25.8 Hz, 1C), 71.7, 57.7, 46.9, 35.8 (d, *J* = 3.4, 1C), 28.8, 27.5, 25.7, 25.3. HRMS-ESI (m/z) calcd for (C_23_H_26_F_2_N_3_O_5_ + H)^+^: 462.1835, found: 462.1817. 

### 4.12. N-(2,4-Difluorobenzyl)-9-hydroxy-2-(3-methoxypropyl)-1,8-dioxo-1,8-dihydro-2H-pyrido [1,2-a]pyrazine-7-carboxamide (***6g***)

White solid, 32% yield, 307 mg from 1 g of **8**, mp 200-203 °C; ^1^H NMR (400 MHz, DMSO-*d*_6_) δ 12.12 (s, 1H), 10.59 (t, *J* = 5.9 Hz, 1H), 8.78 (s, 1H), 7.49 (d, *J* = 6.2 Hz, 1H), 7.46–7.38 (m, 1H), 7.29–7.20 (m, 1H), 7.09–7.04 (m, 1H), 6.91 (d, *J* = 6.2 Hz, 1H), 4.56 (d, *J* = 5.9 Hz, 2H), 3.80 (t, *J* = 7.1 Hz, 2H), 3.37 (t, *J* = 6.1 Hz, 2H), 3.22 (s, 3H), 1.88 (p, *J* = 6.3 Hz, 2H). ^13^C NMR (101 MHz, DMSO-*d*_6_) δ 168.1, 163.4, 161.5 (dd, *J* = 245.5, 12.3 Hz, 1C), 160.7, 160.2 (dd, *J* = 245.5, 12.3 Hz, 1C), 152.9, 134.9, 130.8 (dd, *J* = 9.9, 6.0 Hz, 1C), 122.2 (dd, *J* = 25.3, 3.7 Hz, 1C), 118.9, 117.8, 117.9, 113.0, 111.4 (dd, *J* = 20.9, 3.6 Hz, 1C), 103.8 (t, *J* = 25.8 Hz, 1C), 68.9, 57.9, 44.8, 35.8 (d, *J* = 3.6 Hz, 1C), 27.6. HRMS-ESI (m/z) calcd for (C_20_H_19_F_2_N_3_O_5_ + H)^+^: 420.1366, found: 420.1372.

### 4.13. N-(2,4-Difluorobenzyl)-9-hydroxy-2-(2-methoxyethyl)-1,8-dioxo-1,8-dihydro-2H-pyrido [1,2-a]pyrazine-7-carboxamide (***6h***)

White solid, 33% yield, 151 mg from 500 mg of **8**, mp 250-152 °C; ^1^H NMR (400 MHz, DMSO-*d*_6_) δ 12.01 (s, 1H), 10.57 (t, *J* = 5.9 Hz, 1H), 8.77 (s, 1H), 7.47 (d, *J* = 6.3 Hz, 1 H), 7.49–7.38 (m, 1H), 7.29–7.20 (m, 1H), 7.10–7.03 (m, 1H), 6.89 (d, *J* = 6.3 Hz, 1H), 4.57 (d, *J* = 5.9 Hz, 2H), 3.94 (t, *J* = 5.3 Hz, 2H), 3.60 (t, *J* = 5.3 Hz, 2H), 3.27 (s, 3H). ^13^C NMR (101 MHz, DMSO-*d*_6_) δ 168.1, 163.4, 161.5 (dd, *J* = 245.5, 12.3 Hz, 1C), 160.6, 160.3 (dd, *J* = 247.8, 12.5 Hz, 1C), 153.0, 135.1, 130.8 (dd, *J* = 9.7, 6.0 Hz, 1C), 122.2 (dd, *J* = 15.3, 3.8 Hz, 1C), 119.2, 117.6, 117.3, 112.7, 111.4 (dd, *J* = 21.2, 3.6 Hz, 1C), 103.8 (t, *J* = 25.7 Hz, 1C), 68.9, 58.1, 46.4, 35.9 (d, *J* = 3.5 Hz, 1C). HRMS-ESI (m/z) calcd for (C_18_H_17_F_2_N_3_O_5_ + H)^+^: 406.1209, found: 406.1195.

### 4.14. N-(2,4-Difluorobenzyl)-9-hydroxy-2-(4-methylpentan-2-yl)-1,8-dioxo-1,8-dihydro-2H-pyrido [1,2-a]pyrazine-7-carboxamide (***6i***)

White solid, 36% yield, 180 mg from 500 mg of **8**, mp 199-203 °C; ^1^H NMR (400 MHz, DMSO-*d*_6_) δ 12.24 (s, 1H), 10.59 (t, *J* = 5.9 Hz, 1H), 8.80 (s, 1H), 7.56 (d, *J* = 6.4 Hz, 1H), 7.47–7.38 (m, 1H), 7.30–7.19 (m, 1H), 7.12–7.04 (m, 1H), 7.02 (d, *J* = 6.4 Hz, 1H), 4.96–4.79 (m, 1H), 4.57 (d, *J* = 5.9 Hz, 2H), 1.71 (dd, *J* = 11.0, 7.5 Hz, 1H), 1.41 (dddd, *J* = 13.9, 11.7, 7.2, 3.7 Hz, 2H), 1.26 (d, *J* = 6.8 Hz, 3H), 0.87 (dd, *J* = 9.1, 6.0 Hz, 6H). ^13^C NMR (101 MHz, DMSO-*d*_6_) δ 168.1, 163.4, 161.5 (dd, *J* = 245.7, 12.4 Hz, 1C), 160.5, 160.3 (dd, *J* = 247.4, 12.6 Hz, 1C), 153.1, 134.9, 130.8 (dd, *J* = 9.8, 6.1 Hz, 1C), 122.2 (dd, *J* = 15.4, 3.7 Hz, 1C), 117.6, 117.2, 114.2, 113.8, 111.4 (dd, *J* = 21.2, 3.7 Hz, 1C), 103.8 (t, *J* = 25.8 Hz, 1C), 47.6, 42.5, 35.8 (dd, *J* = 3.6 Hz, 1C), 24.2, 22.7, 21.8, 18.9. HRMS-ESI (m/z) calcd for (C_22_H_23_F_2_N_3_O_4_ + H)^+^: 432.1729, found: 432.1716.

### 4.15. Reduction of Double Bond in the Ring B via Catalytic Hydrogenation: Synthesis of N-(2,4-Difluorobenzyl)-9-hydroxy-2-methyl-1,8-dioxo-1,3,4,8-tetrahydro-2H-pyrido [1,2-a]pyrazine-7-carboxamide (***7a***)

To a 25 mL single-necked round bottom flask was added compound **6a** (50 mg, 0.14 mmol, 1.0 equiv.), which was subjected to the catalytic hydrogenation reaction using 10 mmol% (10% Pd/C, 15 mg, 0.1 equiv.) in a binary mixture of solvents (DMF:MeOH; 2:3, 10 mL) under hydrogen balloon for 20 h. the reaction was the filtered through celite to remove active palladium and repeatedly washed with methanol (4–5 × 5 mL). The filtrate was then concentrated over rotavapor, and crude product was dissolved in DMSO (3 mL) and isolated using Prep-HPLC to afford **7a** as a white solid (13 mg, 26% yield ), mp 247–252 °C; ^1^H NMR (400 MHz, DMSO-*d*_6_) δ 12.57 (s, 1H), 10.44 (t, *J* = 5.9 Hz, 1H), 8.40 (s, 1H), 7.41–7.34 (m, 1H), 7.28–7.19 (m, 1H), 7.11–7.02 (m, 1H), 4.54 (d, *J* = 5.9 Hz, 2H), 4.45–4.31 (m, 2H), 3.82–3.67 (m, 2H), 3.06 (s, 3H). ^13^C NMR (101 MHz, DMSO-*d*_6_) δ 170.2, 163.9, 162.9, 161.5 (dd, *J* = 245.4, 12.2 Hz, 1C), 160.2 (dd, *J* = 247.4, 12.3 Hz, 1C), 154.0, 139.9, 130.7 (dd, *J* = 9.9, 6.3 Hz, 1C), 122.3 (dd, *J* = 15.1, 3.7 Hz, 1C), 117.4, 114.9, 111.3 (dd, *J* = 21.1, 3.7 Hz, 1C), 103.8 (d, *J* = 25.7 Hz, 1C), 48.7, 45.7, 35.7 (d, *J* = 3.6 Hz, 1C), 34.1. HRMS-ESI (m/z) calcd for (C_17_H_15_F_2_N_3_O_4_ + H)^+^: 364.1103, found: 364.1112.

### 4.16. General Procedure II: Synthesis of Saturated Compounds ***7b***–***7i***

The titled compounds were synthesized as per the catalytic hydrogenation reaction procedure used for the compound **7a** and isolated using Prep-HPLC (binary mixture of 0.1% TFA in acetonitrile and 0.1% TFA in H_2_O as eluents).

### 4.17. N-(2,4-Difluorobenzyl)-9-hydroxy-2-isopropyl-1,8-dioxo-1,3,4,8-tetrahydro-2H-pyrido [1,2-a]pyrazine-7-carboxamide (***7b***)

White solid, 78% yield, 55 mg from 70 mg of **6b**, mp 281-287 °C; ^1^H NMR (400 MHz, DMSO-*d*_6_) δ 12.61 (s, 1H), 10.46 (t, *J* = 5.9 Hz, 1H), 8.41 (s, 1H), 7.44–7.36 (m, 1H), 7.24 (m, 1H), 7.11–7.01 (m, 1H), 4.80–4.67 (m, 1H), 4.54 (d, *J* = 5.9 Hz, 2H), 4.41–4.32 (m, 2H), 3.68 (m, 2H), 1.16 (s, 6H). ^13^C NMR (101 MHz, DMSO-*d*_6_) δ 170.2, 163.9, 162.0, 161.4 (dd, *J* = 245.3, 12.2 Hz, 1C), 160.2 (dd, *J* = 247.2, 12.4 Hz, 1C), 154.2, 139.7, 130.7 (dd, *J* = 9.9, 6.2 Hz, 1C), 122.4 (dd, *J* = 15.2, 3.6 Hz, 1C), 117.5, 114.9, 111.3 (dd, *J* = 21.1, 3.6 Hz, 1C), 103.8 (t, *J* = 25.8 Hz, 1C), 49.2, 44.5, 37.7, 35.7, (d, *J* = 3.6 Hz, 1C), 18.7. HRMS-ESI (m/z) calcd for (C_19_H_19_F_2_N_3_O_4_ + H)^+^: 392.1416, found: 392.1426.

### 4.18. N-(2,4-Difluorobenzyl)-9-hydroxy-2-(6-hydroxyhexyl)-1,8-dioxo-1,3,4,8-tetrahydro-2H-pyrido [1,2-a]pyrazine-7-carboxamide (***7c***)

White solid, 27% yield, 55 mg from 200 mg of **6c**, mp 208-210 °C; ^1^H NMR (400 MHz, DMSO-*d*_6_) δ 12.56 (s, 1H), 10.44 (t, *J* = 5.9 Hz, 1H), 8.40 (s, 1H), 7.43–7.35 (m, 1H), 7.29–7.22 (m, 1H), 7.10–7.04 (m, 1H), 4.54 (d, *J* = 5.9 Hz, 2H), 4.41–4.34 (m, 2H), 3.80–3.73 (m, 2H), 3.48 (t, *J* = 7.3 Hz, 2H), 3.38 (t, *J* = 6.4 Hz, 2H), 1.57 (p, *J* = 7.3 Hz, 2H), 1.42 (p, *J* = 6.4 Hz, 2H), 1.36–1.24 (m, 4H). ^13^C NMR (101 MHz, DMSO-*d*_6_) δ 170.2, 163.9, 162.6, 161.5 (dd, *J* = 245.2, 12.3 Hz, 1C), 160.2 (dd, *J* = 247.3, 12.4 Hz, 1C), 154.2, 139.9, 130.7 (dd, *J* = 9.8, 6.1 Hz, 1C), 122.4 (dd, *J* = 15.3, 3.6 Hz, 1C), 117.4, 114.9, 111.3 (dd, *J* = 21.1, 3.6 Hz, 1C), 103.8 (t, *J* = 25.8 Hz, 1C), 60.6, 49.0, 46.4, 43.8, 35.7 (d, *J* = 3.6 Hz, 1C), 32.3, 26.4, 26.0, 25.2. HRMS-ESI (m/z) calcd for (C_22_H_25_F_2_N_3_O_5_ + H)^+^: 450.1835, found: 450.1844.

### 4.19. N-(2,4-Difluorobenzyl)-9-hydroxy-2-(5-hydroxypentyl)-1,8-dioxo-1,3,4,8-tetrahydro-2H-pyrido [1,2-a]pyrazine-7-carboxamide (***7d***)

White solid, 59% yield, 42 mg from 70 mg of **6d**, mp 201-207 °C; ^1^H NMR (400 MHz, DMSO-*d*_6_) δ 12.56 (s, 1H), 10.44 (t, *J* = 5.9 Hz, 1H), 8.40 (s, 1H), 7.43–7.34 (m, 1H), 7.27–7.18 (m, 1H), 7.10–7.02 (m, 1H), 4.53 (d, *J* = 5.9 Hz, 2H), 4.42–4.32 (m, 2H), 3.78–3.73 (m, 2H), 3.48 (t, *J* = 7.2 Hz, 2H), 3.38 (t, *J* = 6.4 Hz, 2H), 1.58 (hept, *J* = 8.8, 7.9 Hz, 2H), 1.44 (dt, *J* = 14.2, 6.7 Hz, 2H), 1.35–1.22 (m, 2H). ^13^C NMR (101 MHz, DMSO-*d*_6_) δ 170.2, 163.9, 162.6, 161.5 (dd, *J* = 245.3, 12.3 Hz, 1C), 160.2 (dd, *J* = 247.4, 12.4 Hz, 1C), 154.2, 139.9, 130.7 (dd, *J* = 9.9, 6.2 Hz, 1C), 122.4 (dd, *J* = 15.3, 3.6 Hz, 1C), 117.4, 114.9, 111.3 (dd, *J* = 21.1, 3.8 Hz, 1C), 103.8 (t, *J* = 25.6 Hz, 1C), 60.5, 49.0, 46.4, 43.8, 35.7 (d, *J* = 3.5 Hz, 1C), 32.1, 26.2, 22.7. HRMS-ESI (m/z) calcd for (C_21_H_23_F_2_N_3_O_5_ + H)^+^: 436.1679, found: 436.1686.

### 4.20. N-(2,4-Difluorobenzyl)-9-hydroxy-2-(4-hydroxybutyl)-1,8-dioxo-1,3,4,8-tetrahydro-2H-pyrido [1,2-a]pyrazine-7-carboxamide (***7e***)

White solid, 63% yield, 16 mg from 25 mg of **6e**, mp 210-215 °C; ^1^H NMR (400 MHz, DMSO-*d*_6_) δ 12.56 (s, 1H), 10.44 (t, *J* = 5.9 Hz, 1H), 8.40 (s, 1H), 7.44–7.34 (m, 1H), 7.29–7.20 (m, 1H), 7.11–7.02 (m, 1H), 4.54 (d, *J* = 5.9 Hz, 2H), 4.43–4.32 (m, 2H), 3.80–3.71 (m, 2H), 3.50 (t, *J* = 7.3 Hz, 2H), 3.42 (t, *J* = 6.3 Hz, 2H), 1.69–1.55 (m, 2H), 1.44 (dt, *J* = 8.8, 6.4 Hz, 2H). ^13^C NMR (101 MHz, DMSO-*d*_6_) δ 170.2, 163.9, 162.6, 161.4 (dd, *J* = 245.4, 12.1 Hz, 1C), 160.2 (dd, *J* = 247.4, 12.5 Hz, 1C) 154.2, 139.9, 130.7 (dd, *J* = 9.9, 6.2 Hz, 1C), 122.3 (dd, *J* = 15.3, 3.6 Hz, 1C), 117.4, 114.9, 111.3 (dd, *J* = 21.1, 3.6 Hz, 1C), 103.8 (t, *J* = 25.9 Hz, 1C), 60.2, 49.0, 46.3, 43.8, 35.7 (d, *J* = 3.5 Hz, 1C), 29.6, 23.1. HRMS-ESI (m/z) calcd for (C_20_H_21_F_2_N_3_O_5_ + H)^+^: 422.1522, found: 422.1528.

### 4.21. N-(2,4-Difluorobenzyl)-9-hydroxy-2-(6-methoxyhexyl)-1,8-dioxo-1,3,4,8-tetrahydro-2H-pyrido [1,2-a]pyrazine-7-carboxamide (***7f***)

White solid, 50% yield, 35 mg from 70 mg of **6f**. mp 228-232 °C; ^1^H NMR (400 MHz, DMSO-*d*_6_) δ 12.56 (s, 1H), 10.44 (t, *J* = 5.9 Hz, 1H), 8.40 (s, 1H), 7.44–7.35 (m, 1H), 7.28–7.19 (m, 1H), 7.10–7.01 (m, 1H), 4.54 (d, *J* = 5.9 Hz, 2H), 4.43–4.31 (m, 2H), 3.83–3.69 (m, 2H), 3.48 (t, *J* = 7.3 Hz, 2H), 3.29 (t, *J* = 6.5 Hz, 2H), 3.20 (s, 3H), 1.57 (p, *J* = 7.2 Hz, 2H), 1.49 (p, *J* = 6.8 Hz, 2H), 1.31 (h, *J* = 6.1 Hz, 4H). ^13^C NMR (101 MHz, DMSO-*d*_6_) δ 170.2, 163.9, 162.6, 161.4 (dd, *J* = 245.4, 12.4 Hz, 1C), 160.3 (dd, *J* = 247.2, 12.4 Hz, 1C), 154.2, 139.9, 130.7 (dd, *J* = 9.8, 6.1 Hz, 1C), 122.4 (dd, *J* = 15.3, 3.7 Hz, 1C), 117.4, 114.9, 111.3 (dd, *J* = 21.1, 3.6 Hz, 1C), 103.8 (t, *J* = 25.7 Hz, 1C), 71.8, 57.5, 49.0, 46.3, 43.8, 35.7 (d, *J* = 3.6 Hz, 1C), 28.9, 26.3, 25.9, 25.3. HRMS-ESI (m/z) calcd for (C_23_H_27_F_2_N_3_O_5_ + H)^+^: 464.1992, found: 464.1977.

### 4.22. N-(2,4-Difluorobenzyl)-9-hydroxy-2-(3-methoxypropyl)-1,8-dioxo-1,3,4,8-tetrahydro-2H-pyrido [1,2-a]pyrazine-7-carboxamide (***7g***)

White solid, 56% yield, 90 mg from 150 mg of **6g**, mp 250-252 C; ^1^H NMR (400 MHz, DMSO-*d*_6_) δ 12.53 (s, 1H), 10.44 (t, *J* = 5.9 Hz, 1H), 8.40 (s, 1H), 7.42–7.38 (m, 1H), 7.28–7.19 (m, 1H), 7.12–6.99 (m, 1H), 4.54 (d, *J* = 5.9 Hz, 2H), 4.44–4.32 (m, 2H), 3.80–3.71 (m, 2H), 3.54 (t, *J* = 7.1 Hz, 2H), 3.37 (t, *J* = 6.1 Hz, 2H), 3.23 (s, 3H), 1.82 (p, *J* = 6.3 Hz, 2H). ^13^C NMR (101 MHz, DMSO-*d*_6_) δ 170.1, 163.9, 162.7, 161.5 (dd, *J* = 245.2, 12.0 Hz, 1C), 160.2 (dd, *J* = 247.4, 12.4 Hz, 1C), 154.1, 139.9, 130.7 (dd, *J* = 9.8, 6.1 Hz, 1C), 122.4 (dd, *J* = 15.3, 3.7 Hz, 1C), 117.4, 114.9, 111.3 (dd, *J* = 21.1, 3.6 Hz, 1C), 103.8 (t, *J* = 25.8 Hz, 1C), 69.4, 57.9, 49.0, 44.2, 44.1, 35.7 (d, *J* = 3.5 Hz, 1C), 26.6. HRMS-ESI (m/z) calcd for (C_20_H_21_F_2_N_3_O_5_ + H)^+^: 422.1522, found: 422.1528.

### 4.23. N-(2,4-Difluorobenzyl)-9-hydroxy-2-(2-methoxyethyl)-1,8-dioxo-1,3,4,8-tetrahydro-2H-pyrido [1,2-a]pyrazine-7-carboxamide (***7h***)

White solid, 54% yield, 55 mg from 100 mg of **6h**, mp 207-210 °C; ^1^H NMR (400 MHz, DMSO-*d*_6_) δ 12.43 (s, 1H), 10.43 (t, *J* = 5.9 Hz, 1H), 8.39 (s, 1H), 7.44–7.34 (m, 1H), 7.28–7.19 (m, 1H), 7.11–7.02 (m, 1H), 4.54 (d, *J* = 5.9 Hz, 2H), 4.41–4.29 (m, 2H), 3.82–3.77 (m, 2H), 3.68 (t, *J* = 5.3 Hz, 2H), 3.56 (d, *J* = 5.3 Hz, 2H), 3.27 (s, 3H). ^13^C NMR (101 MHz, DMSO-*d*_6_) δ 170.1, 163.8, 162.7, 161.4 (dd, *J* = 245.1, 12.1 Hz, 1C), 160.3 (dd, *J* = 247.3, 12.0 Hz, 1C), 154.2, 140.0, 130.8 (dd, *J* = 9.8, 6.1 Hz, 1C), 122.4 (dd, *J* = 15.4, 3.6 Hz, 1C), 117.3, 114.9, 111.3 (dd, *J* = 21.1, 3.6 Hz, 1C), 103.8 (t, *J* = 25.8 Hz, 1C), 68.9, 58.1, 49.1, 46.0, 44.8, 35.7 (d, *J* = 3.6 Hz, 1C). HRMS-ESI (m/z) calcd for (C_19_H_19_F_2_N_3_O_5_ + H)^+^: 408.1366, found: 408.1349.

### 4.24. N-(2,4-Difluorobenzyl)-9-hydroxy-2-(4-methylpentan-2-yl)-1,8-dioxo-1,3,4,8-tetrahydro-2H-pyrido [1,2-a]pyrazine-7-carboxamide (***7i***)

White solid, 85% yield, 85 mg from 100 mg of **6i**, mp190-192 °C; ^1^H NMR (400 MHz, DMSO-*d*_6_) δ 12.59 (s, 1H), 10.45 (t, *J* = 5.9 Hz, 1H), 8.41 (s, 1H), 7.45–7.33 (m, 1H), 7.27–7.20 (m, 1H), 7.10–7.02 (m, 1H), 4.82–4.68 (m, 1H), 4.54 (d, *J* = 5.9 Hz, 2H), 4.45–4.24 (m, 2H), 3.74–3.55 (m, 2H), 1.56 (ddd, *J* = 14.0, 9.7, 5.0 Hz, 1H), 1.51–1.38 (m, 1H), 1.24 (ddd, *J* = 13.8, 8.7, 5.3 Hz, 1H), 1.14 (d, *J* = 6.7 Hz, 3H), 0.88 (t, *J* = 6.7 Hz, 6H). ^13^C NMR (101 MHz, DMSO-*d*_6_) δ 170.2, 163.9, 162.4, 161.4 (dd, *J* = 245.4, 12.4 Hz, 1C), 160.2 (dd, *J* = 247.3, 12.4 Hz, 1C), 154.3, 139.8, 130.7 (dd, *J* = 9.8, 6.1 Hz, 1C), 122.4 (dd, *J* = 15.3, 3.6 Hz, 1C), 117.5, 114.9, 111.3 (dd, *J* = 20.9, 3.6 Hz, 1C), 103.8 (t, *J* = 25.9 Hz, 1C), 49.2, 46.5, 41.4, 37.7, 35.7 (d, *J* = 3.4 Hz, 1C), 24.4, 22.9, 21.9, 17.5. HRMS-ESI (m/z) calcd for (C_22_H_25_F_2_N_3_O_4_ + H)^+^: 434.1886, found: 434.1872.

### 4.25. Determination of Antiviral Potencies and Cellular Cytotoxicities

Human embryonic kidney cell culture cell line 293 was acquired from the American Type Culture Collection (ATCC). The human osteosarcoma cell line, HOS, was obtained from Dr. Richard Schwartz (Michigan State University, East Lansing, MI, USA) and grown in Dulbecco’s modified Eagle’s medium (Invitrogen, Carlsbad, CA, USA) supplemented with 5% (*v*/*v*) fetal bovine serum, 5% newborn calf serum, and penicillin (50 units/mL) plus streptomycin (50 µg/mL; Quality Biological, Gaithersburg, MD, USA). The transfection vector, pNLNgoMIVR-ΔLUC was made from pNLNgoMIVR-ΔEnv.HSA by removing the HSA reporter gene and replacing it with a luciferase reporter gene between the NotI and XhoI restriction sites. To produce the new IN mutant S230N used in this study, the IN open reading frame was removed from pNLNgoMIVR-Δ*E*NV.LUC by digestion with *Kpn*I and *Sal*I, and the resulting fragment was inserted between the *Kpn*I and *Sal*I sites of pBluescript KS+. Using that construct as the wild-type template, we prepared the following HIV-1 IN mutant S230N using the QuikChange II XL site-directed mutagenesis kit (Agilent Technologies, Santa Clara, CA, USA) protocol. The following sense oligonucleotides were used with matching cognate antisense oligonucleotides (not shown) (Integrated DNA Technologies, Coralville, IA, USA) in the mutagenesis: S230N 5′-CGGGTTTATTACAGGGACAA**C**AGAGATCCAGTTTGGAAA-3′. The DNA sequence of the IN mutant S230N construct was verified independently by DNA sequence determination. The mutated IN coding sequences from pBluescript KS+ were then subcloned into pNLNgoMIVR-Δ*E*nv.LUC (between the *Kpn*I and *Sal*I sites) to produce mutant HIV-1 constructs, which were also checked by DNA sequencing. VSV-g-pseudotyped HIV was produced by transfections of 293 cells as mentioned earlier. On the day prior to transfection, 293 cells were plated on 100 mm diameter dishes at a density of 1.5 × 10^6^ cells per plate. Next, 293 cells were transfected with 16 µg of pNLNgoMIVR^-^ΔLUC and 4 µg of pHCMV-g (obtained from Dr. Jane Burns, University of California, San Diego) using the calcium phosphate method. At approximately 6 h after the calcium phosphate precipitate was added, 293 cells were washed twice with phosphate-buffered saline (PBS) and incubated with fresh media for 48 h. The virus-containing supernatants were then harvested, clarified by low-speed centrifugation, filtrated, and diluted for preparation in antiviral infection assays. On the day prior to the screen, HOS cells were seeded in a 96-well luminescence cell culture plate at a density of 4000 cells in 100 µL per well. On the day of the screen for cellular cytotoxicity determination, cells were treated with compounds from a concentration range of 250 µM to 0.05 µM and then incubated at 37 °C for 48 h. On the day of the screen for antiviral activity infection assays, cells were treated with compounds from a concentration range of 5 µM to 0.0001 µM using 11 serial dilutions and then incubated at 37 °C for 3 h. After compound incorporation and activation in the cell, 100 µL of virus-stock (WT or mutant) diluted to achieve a luciferase signal between 0.2 and 1.5 Relative Luciferase Units (RLUs) was added to each well and further incubated at 37 °C for 48 h. Cellular cytotoxicity was measured by using the ATP Lite Luminescence detection system and monitored by adding 50 µL of cell lysis buffer from the Luminescence ATP detection assay to each well followed by mixing at 700 rpm at room temperature for 5 min using a compact thermomixer. After addition of 50 µL of reconstituted Luminescence ATP detection assay reagent to all wells except for the negative control/background wells, the plates were mixed at 700 rpm at room temperature for 5 min using a compact thermomixer, incubated at room temperature for 20 min to allow time for signal development, and finally, cytotoxicity was determined using the microplate reader. Infectivity was measured by using the Steady-lite plus luminescence reporter gene assay system (PerkinElmer, Waltham, MA, USA). Luciferase activity was measured by adding 100 µL of Steady-lite plus buffer (PerkinElmer) to the cells, incubating at room temperature for 20 min, and measuring luminescence using a microplate reader. Both cytotoxicity and antiviral activity were normalized to the cellular cytotoxicity and infectivity in cells that featured the absence of target compounds, respectively. KaleidaGraph (Synergy Software, Reading, PA) was used to perform non-linear regression analysis on the data. EC_50_ and CC_50_ values were determined from the fit model [31].

## Figures and Tables

**Table 1:** Antiviral Potencies in Cells Infected with HIV-1 Vectors That Carry WT IN Mutants *^i^*.

R	No	EC_50_ (nM) *^ii^*	CC_50_ (µM)	No	EC_50_ (nM)	CC_50_ (µM)	Ratio *^iii^*
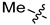	**6a**	2.7 ± 0.2	120.8 ± 2.7	**7a**	4.6 ± 0.7	>250	1.7
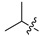	**6b**	3.8 ± 0.6	>250	**7b**	3.0 ± 0.1	162.7 ± 8.0	0.79
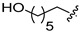	**6c**	4.8 ± 0.9	>250	**7c**	31.7 ± 3.8	>250	6.6
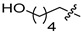	**6d**	10.8 ± 1.7	>250	**7d**	54.5 ± 3.0	149.9 ± 3.9	5.0
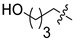	**6e**	16.6 ± 2.0	>250	**7e**	75.7 ± 7.4	>250	4.6
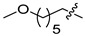	**6f**	4.8 ± 0.2	>250	**7f**	4.6 ± 0.4	>250	0.96
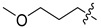	**6g**	2.0 ± 0.2	>250	**7g**	4.0 ± 0.7	>250	2.0
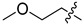	**6h**	1.9 ± 0.4	>250	**7h**	3.9 ± 0.5	138.5 ± 8.5	2.1
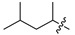	**6i**	4.2 ± 1.0	>250	**7i**	6.0 ± 1.2	>250	1.4

*^i^* EC_50_ values obtained from cells infected with lentiviral vector that has either a WT or the indicated IN mutants; CC_50_ values cytotoxic concentration resulting in 50% reduction in the level of ATP in human osteosarcoma (HOS) cells. *^ii^* EC_50_ values for DTG, BIC, and CAB are 1.6 ± 0.9 nM, 1.9 ± 0.3 nM, and 2.4 ± 0.2 nM, respectively. *^iii^* Ratio of the antiviral potencies after and before hydrogenation.

**Table 2:** Antiviral Potencies in Cells Infected with HIV-1 Vectors Bearing the S230N IN Mutant *^i^*.

R	No	EC_50_ (nM)	No	EC_50_ (nM)	Ratio *^ii^*
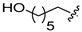	**6c**	WT 4.8 ± 0.9 S230N 9.3 ± 1.9	**7c**	WT 31.7 ± 2.8 S230N 38.0 ± 3.8	6.64.08
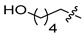	**6d**	WT 10.8 ± 1.7 S230N 15.4 ± 0.3	**7d**	WT 54.5 ± 3.0 S230N 97.0 ± 16.9	5.06.29
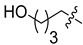	**6e**	WT 16.6 ± 2.0 S230N 30.7 ± 1.4	**7e**	WT 75.7 ± 7.4 S230N 100.8 ± 17.5	4.63.28

*^i^* EC_50_ values obtained from cells infected with lentiviral vector in which IN contains the S230N mutant. *^ii^* Ratio of the antiviral potencies after and before hydrogenation.

## Data Availability

Data is available on request.

## Supplementary Materials

NMR spectra of the synthesized compounds are available in the supporting information: https://www.mdpi.com/article/10.3390/molecules28031428/s1.
