# Supplementary material for: A Practical Approach to Bicyclic Carbamoyl Pyridones with Application to the Synthesis of HIV-1 Integrase Strand Transfer Inhibitors
Source: Molecules. 2023 Feb 2;28(3):1428. doi: 10.3390/molecules28031428 (PMC9919513; doi:10.3390/molecules28031428)

Index

NMR spectra of (compound number)	Page no.
**10**	S3
**13**	S4
**14**	S5
**17**	S6
**18**	S7
**19**	S8
**20**	S9
**21**	S10
**22**	S11
**23**	S12
**24**	S13

NMR spectra of (compound number)	Page no
**25**	S14
**26**	S15
**27**	S16
**28**	S17
**29**	S18
**30**	S19
**31**	S20
**32**	S21
**33**	S22

^{1}H NMR (400 MHz, $CDCl_3$)

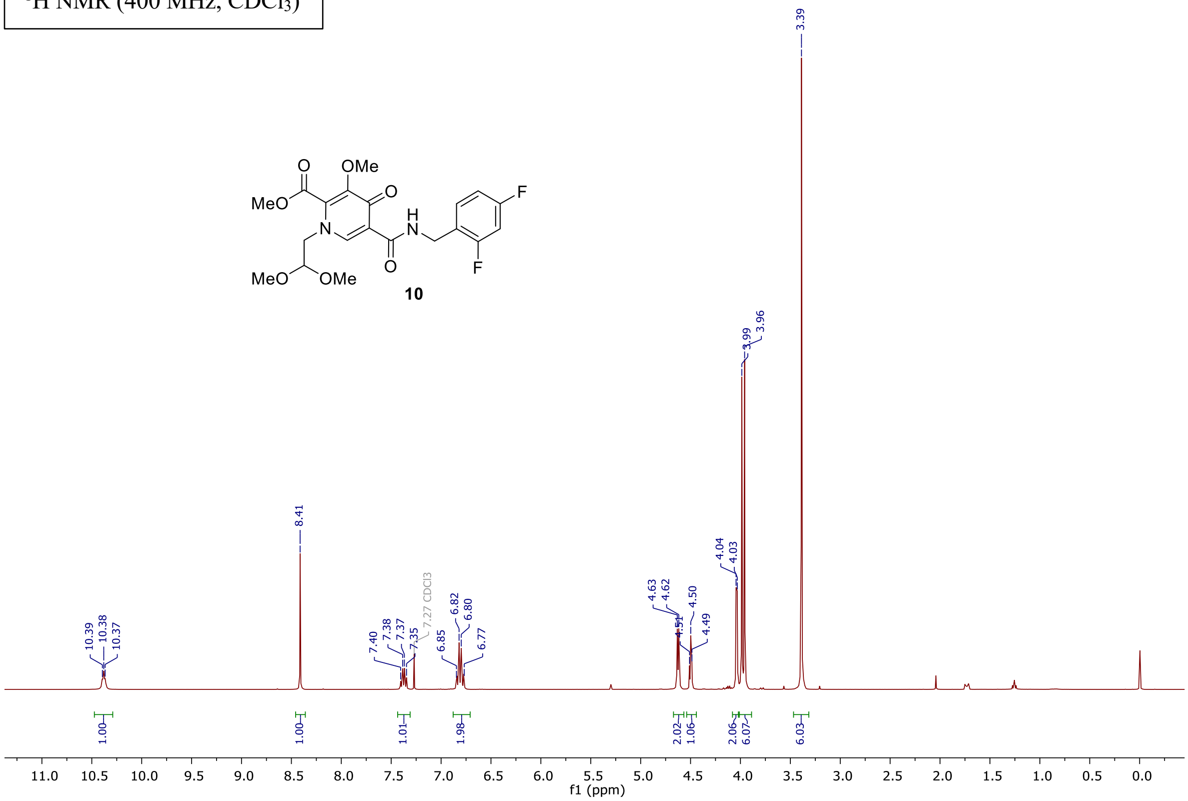

^{13}C NMR (101 MHz, $CDCl_3$)

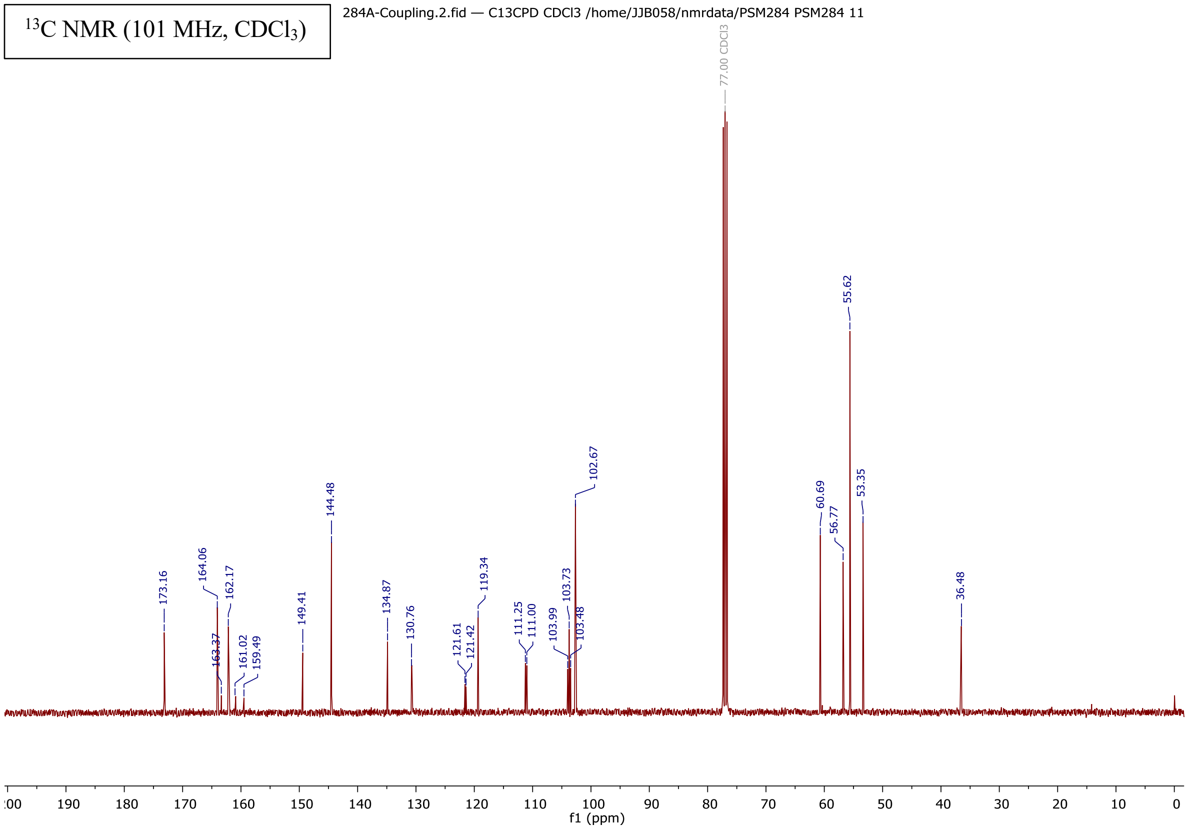

^{1}H NMR (400 MHz, $CDCl_3$)

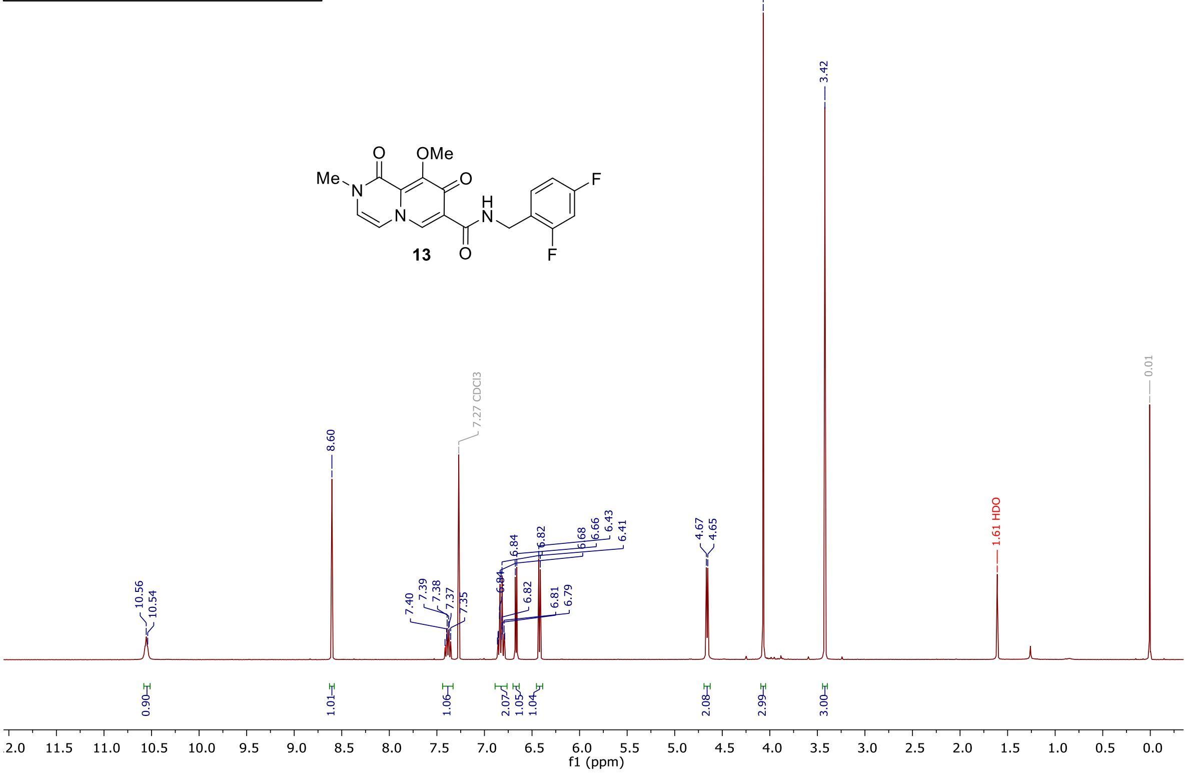

^{13}C NMR (101 MHz, $CDCl_3$)

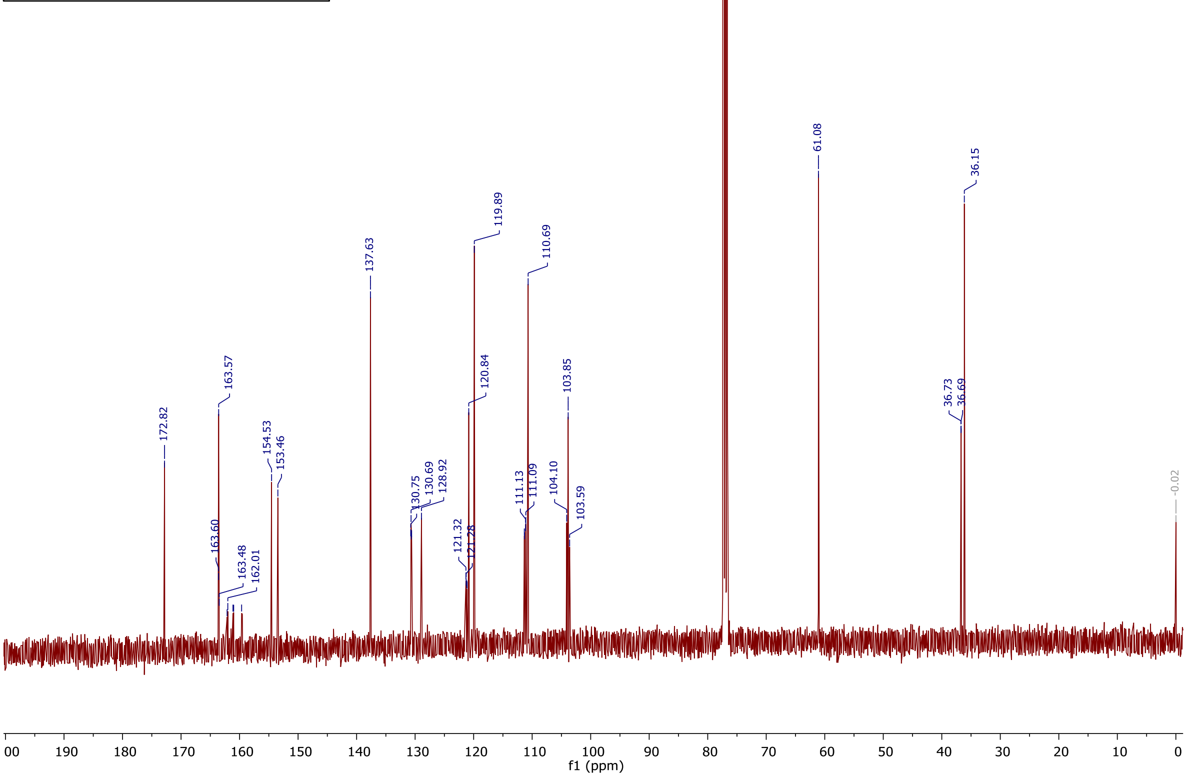

^{1}H NMR (400 MHz, DMSO-d_6)

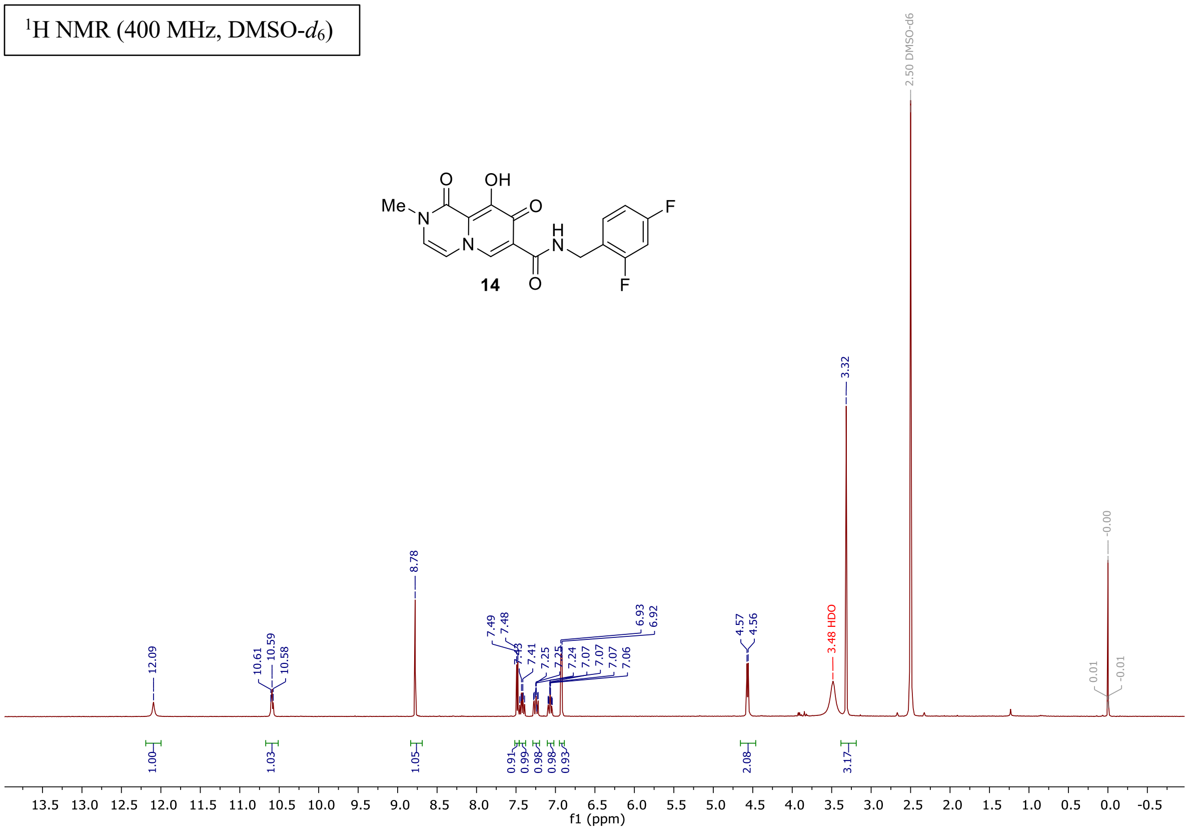

^{13}C NMR (101 MHz, DMSO-d_6)

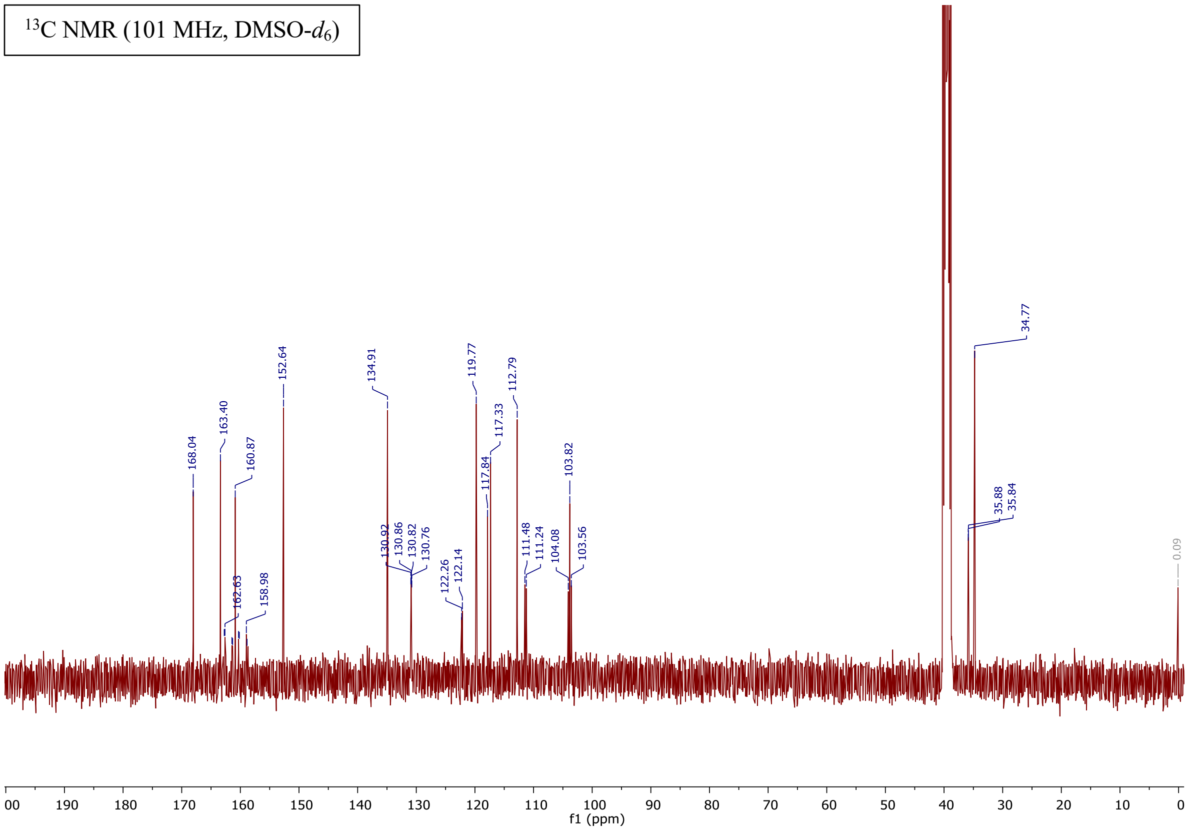

^{1}H NMR (400 MHz, DMSO-d_6)

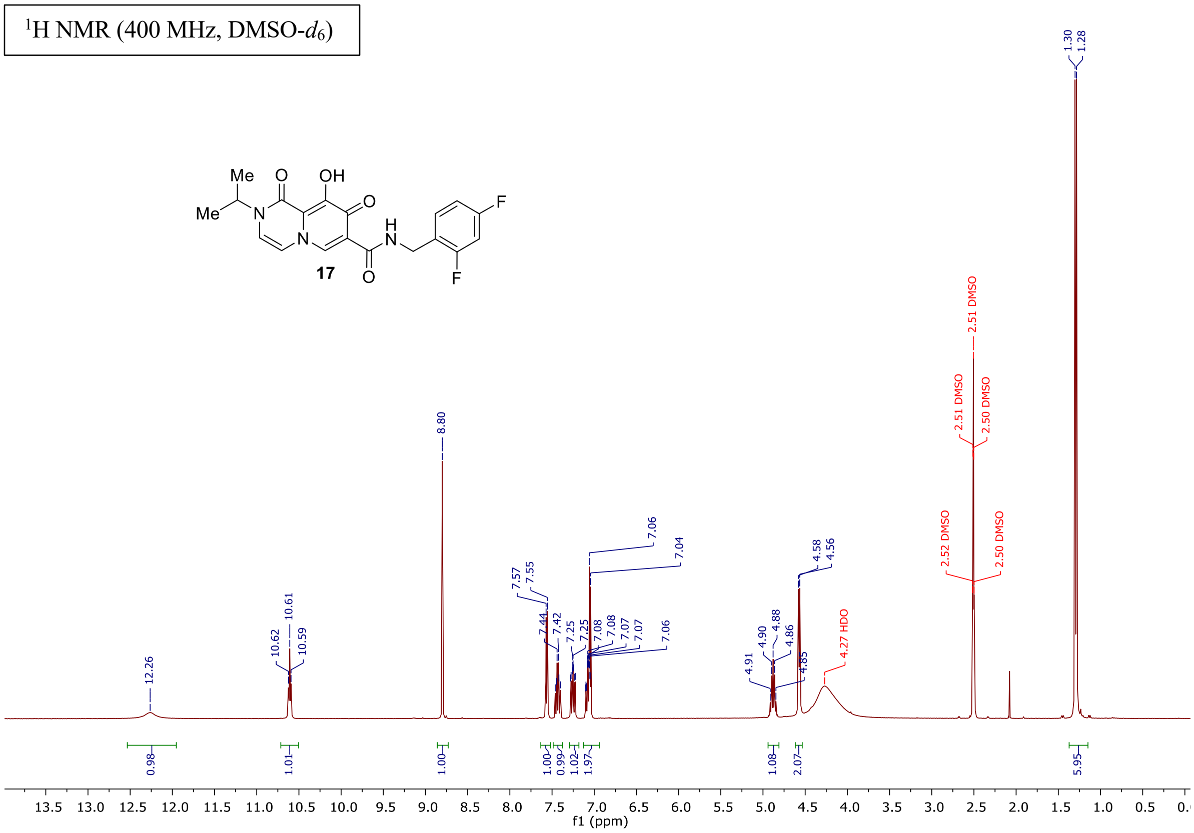

^{13}C NMR (101 MHz, DMSO-d_6)

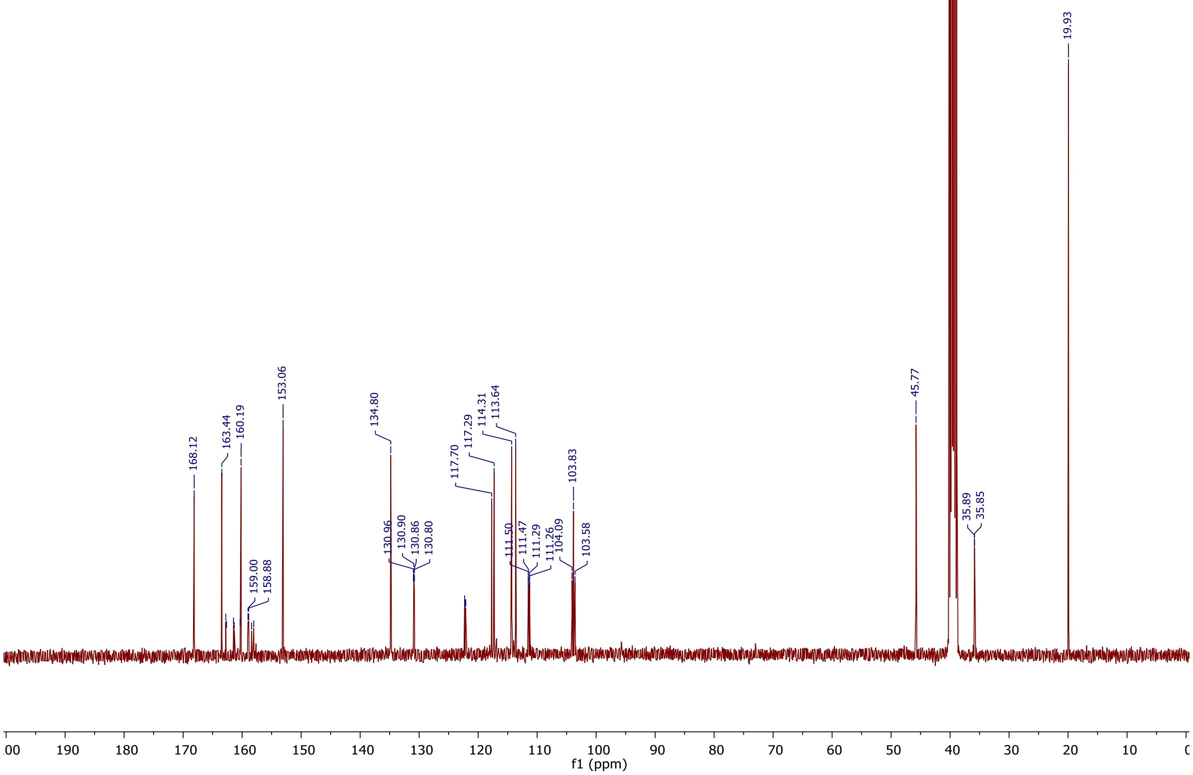

^{1}H NMR (400 MHz, DMSO-d_6)

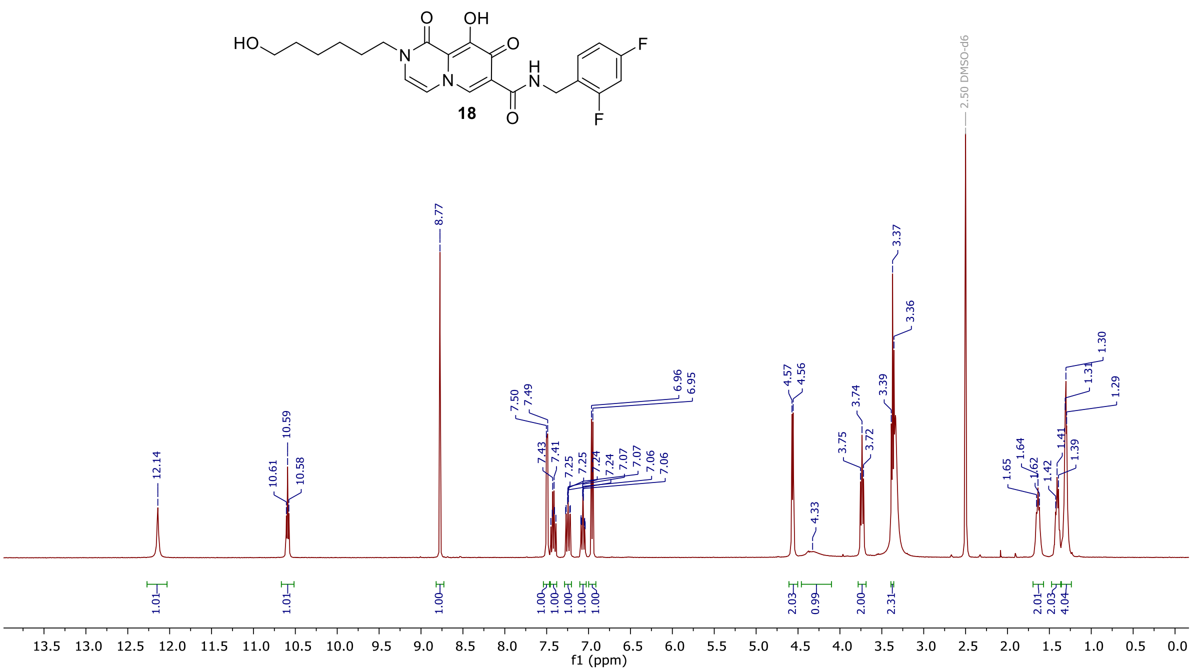

^{13}C NMR (101 MHz, DMSO-d_6)

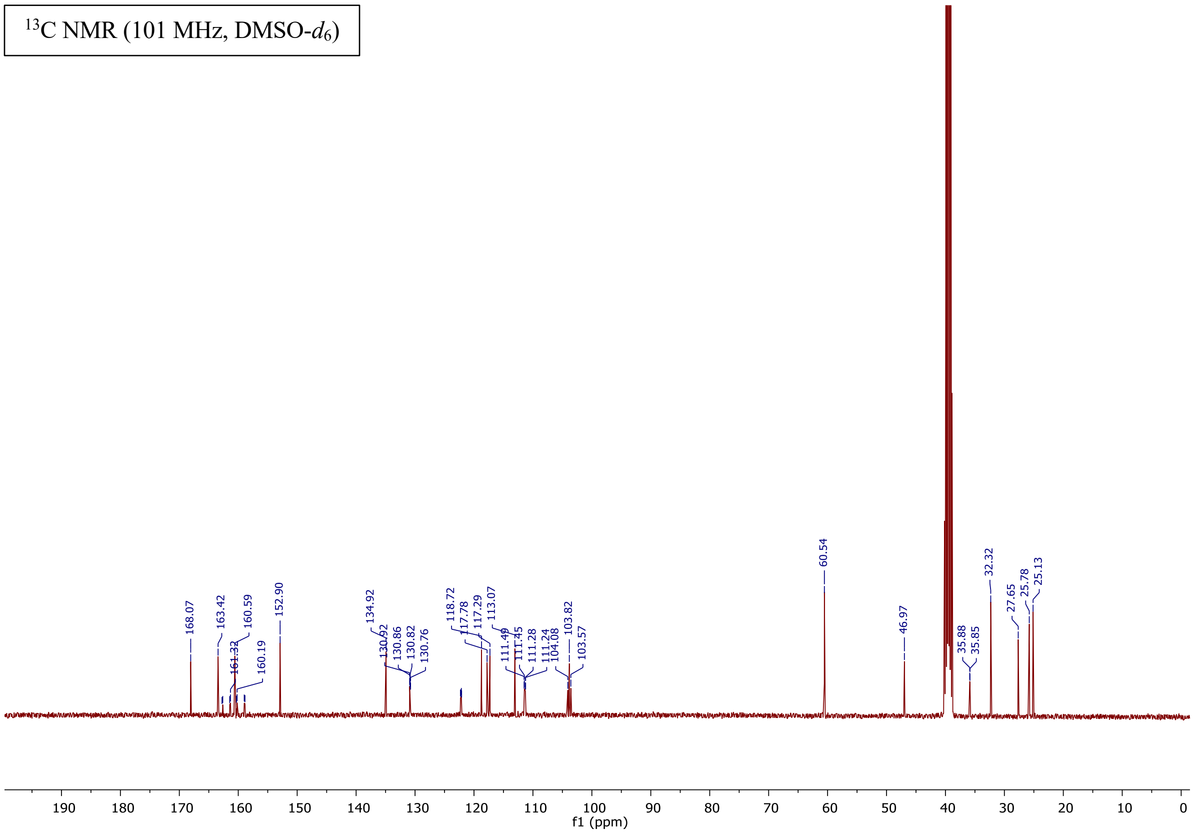

^{1}H NMR (400 MHz, DMSO-d_6)

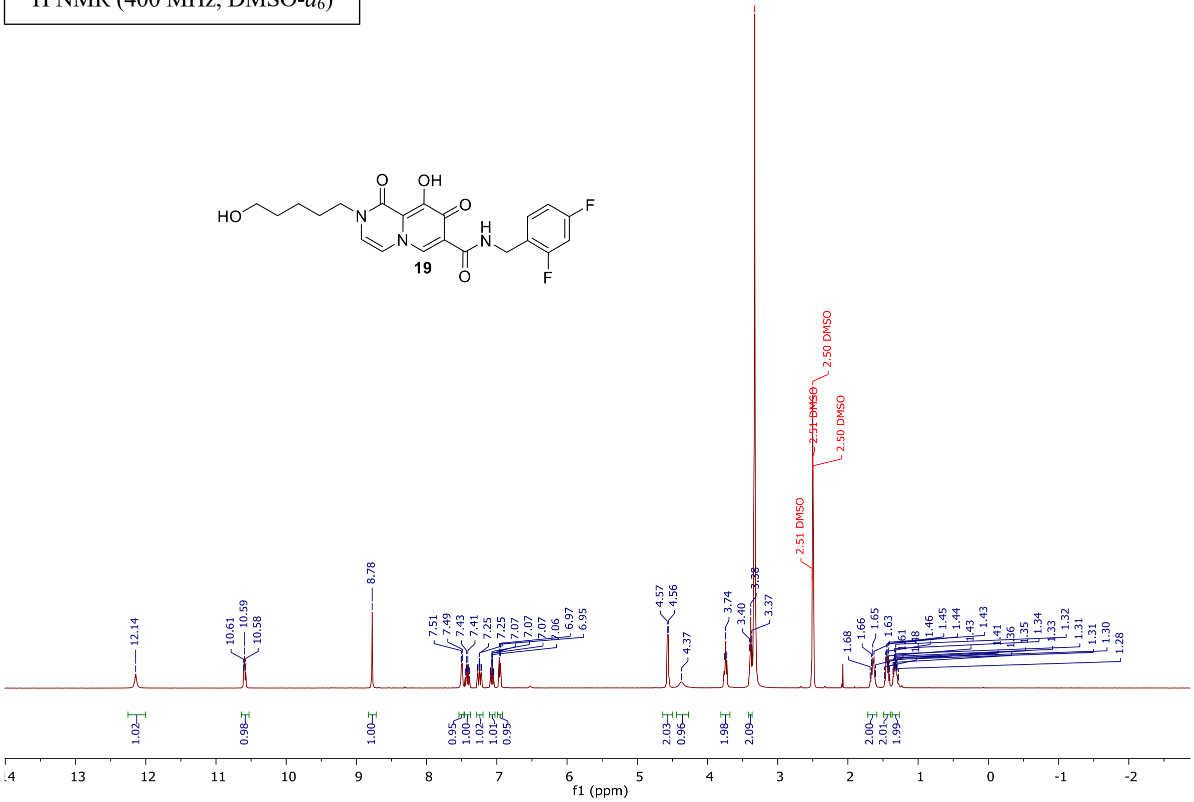

^{13}C NMR (101 MHz, DMSO-d_6)

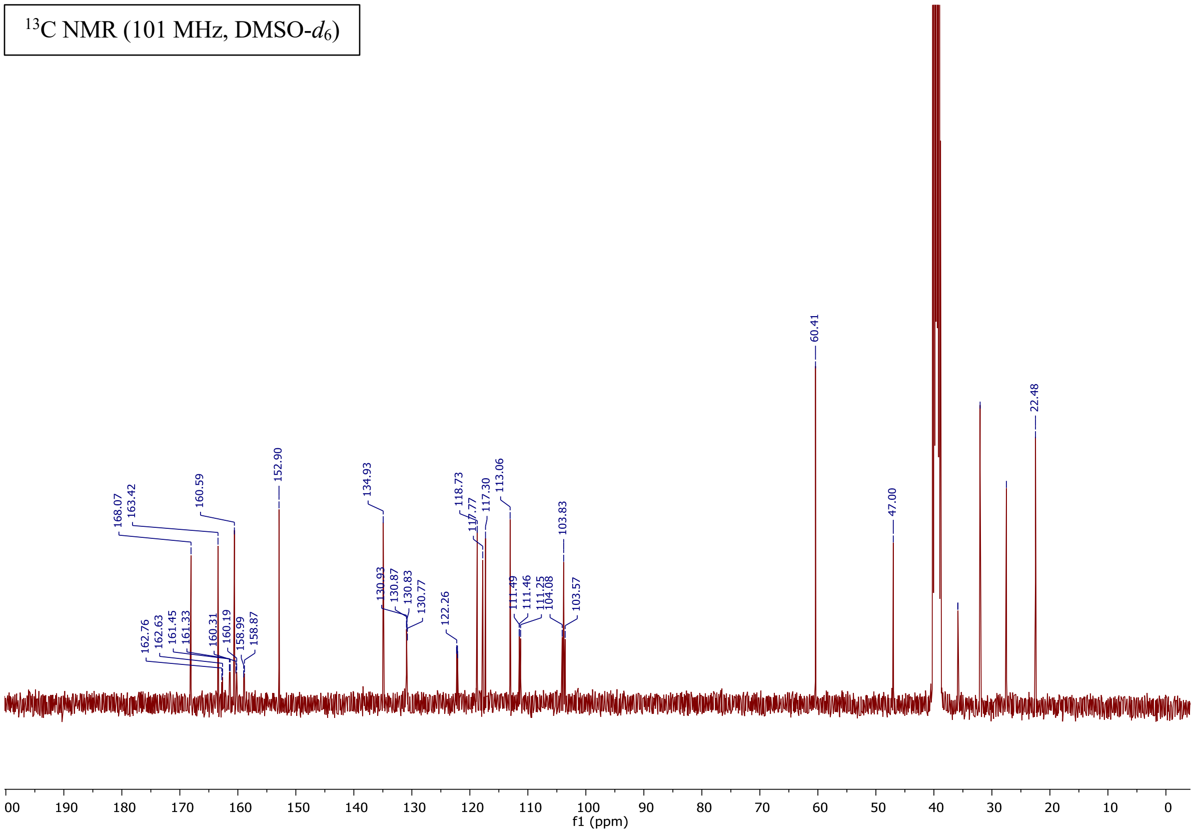

^{1}H NMR (400 MHz, DMSO-d_6)

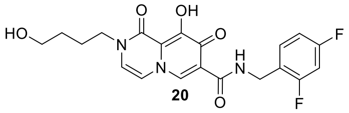

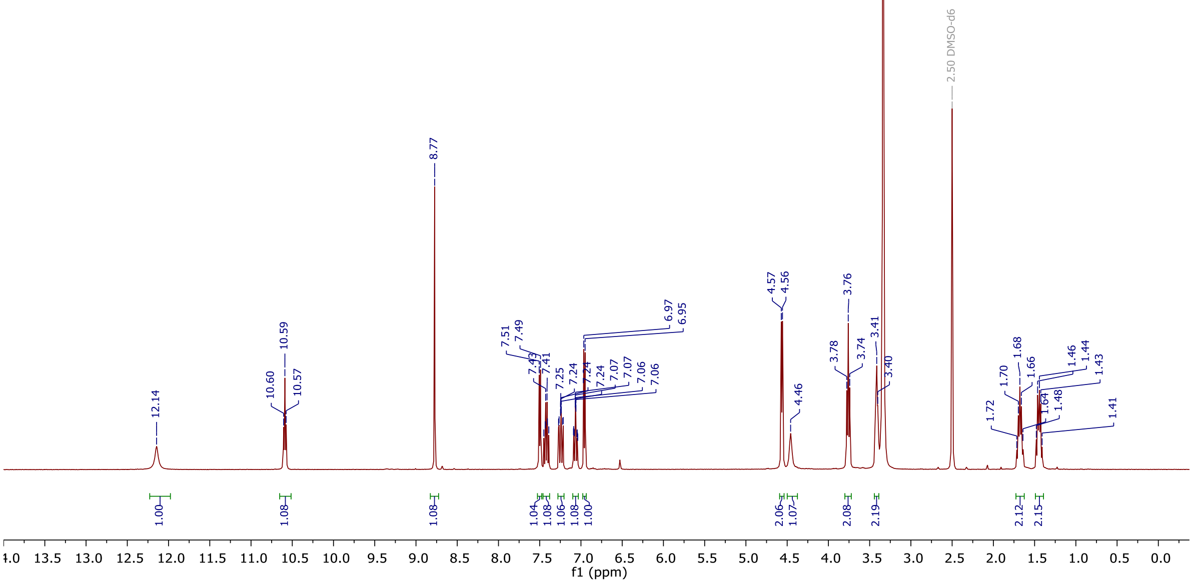

^{13}C NMR (101 MHz, DMSO-d_6)

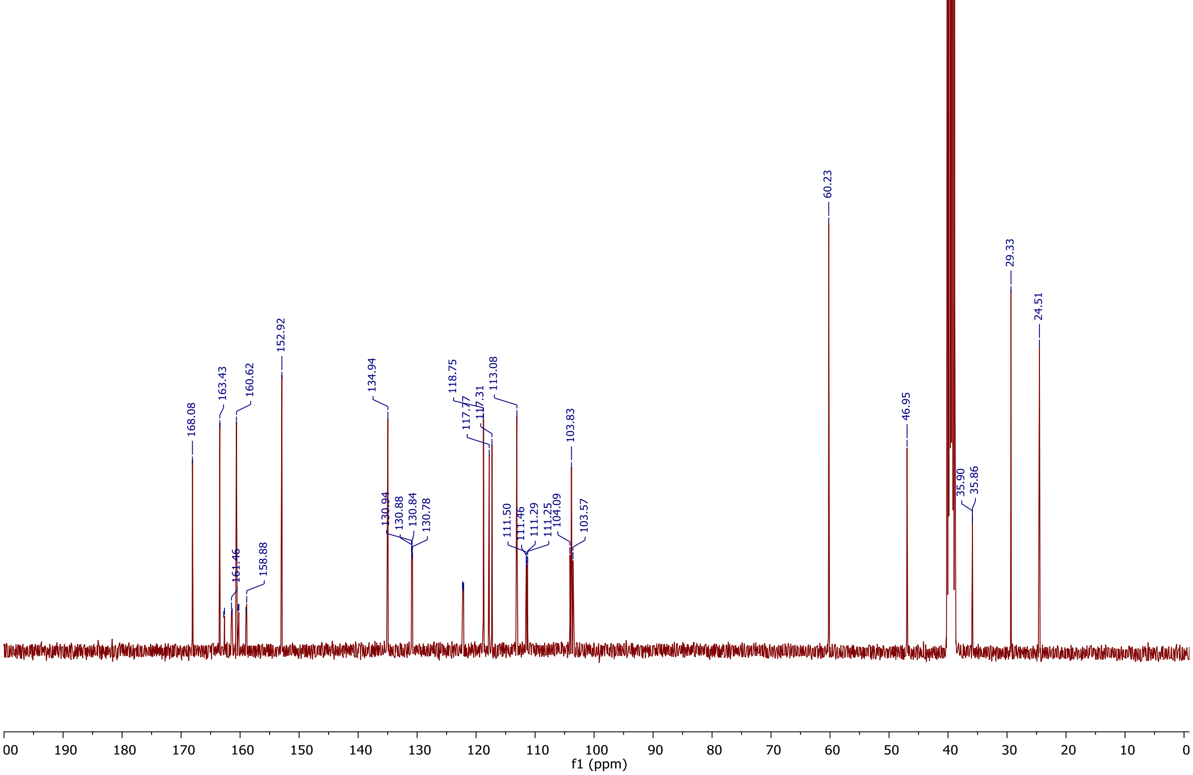

^{1}H NMR (400 MHz, DMSO-d_6)

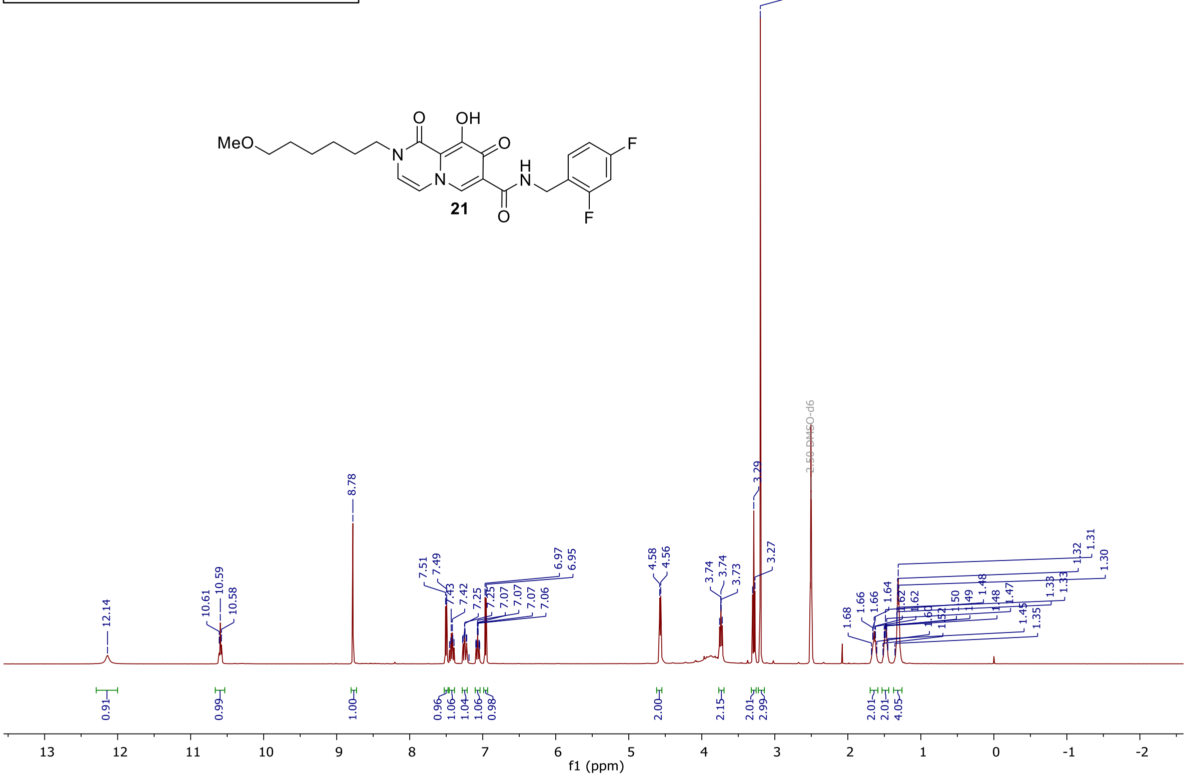

^{13}C NMR (101 MHz, DMSO-d_6)

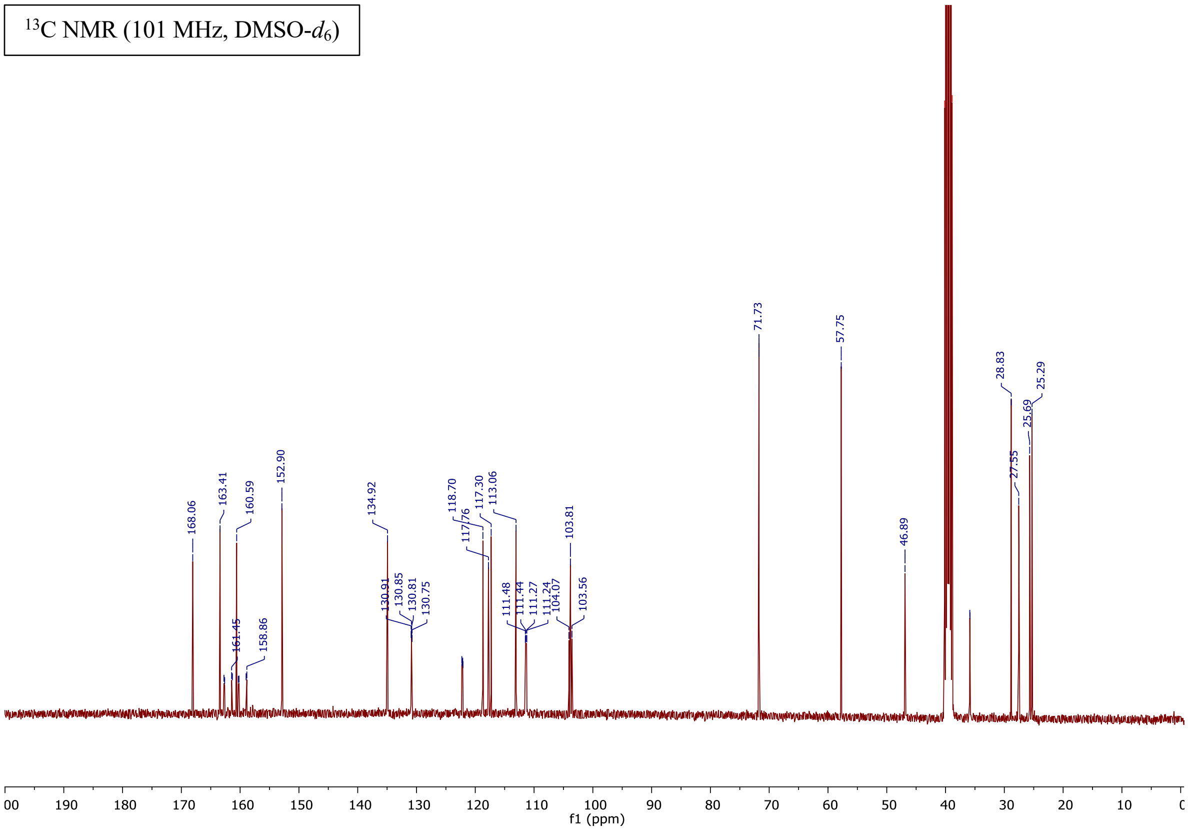

^{1}H NMR (400 MHz, DMSO-d_6)

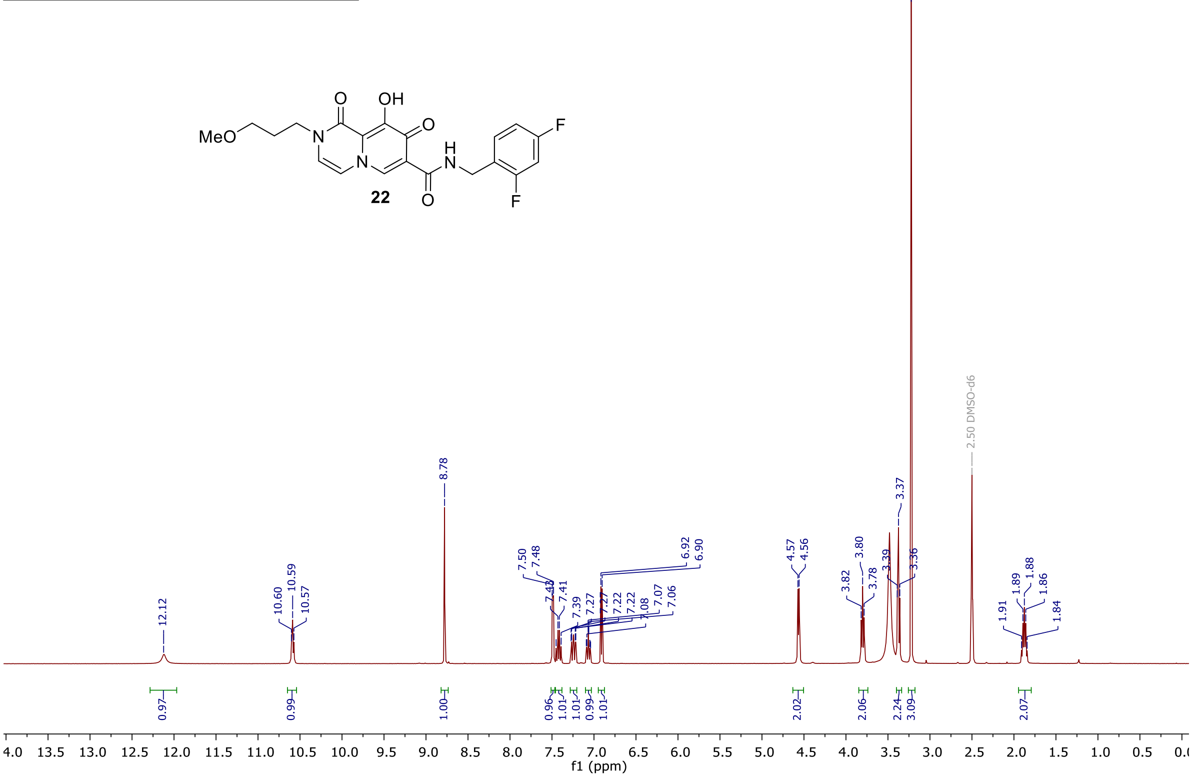

^{13}C NMR (101 MHz, DMSO-d_6)

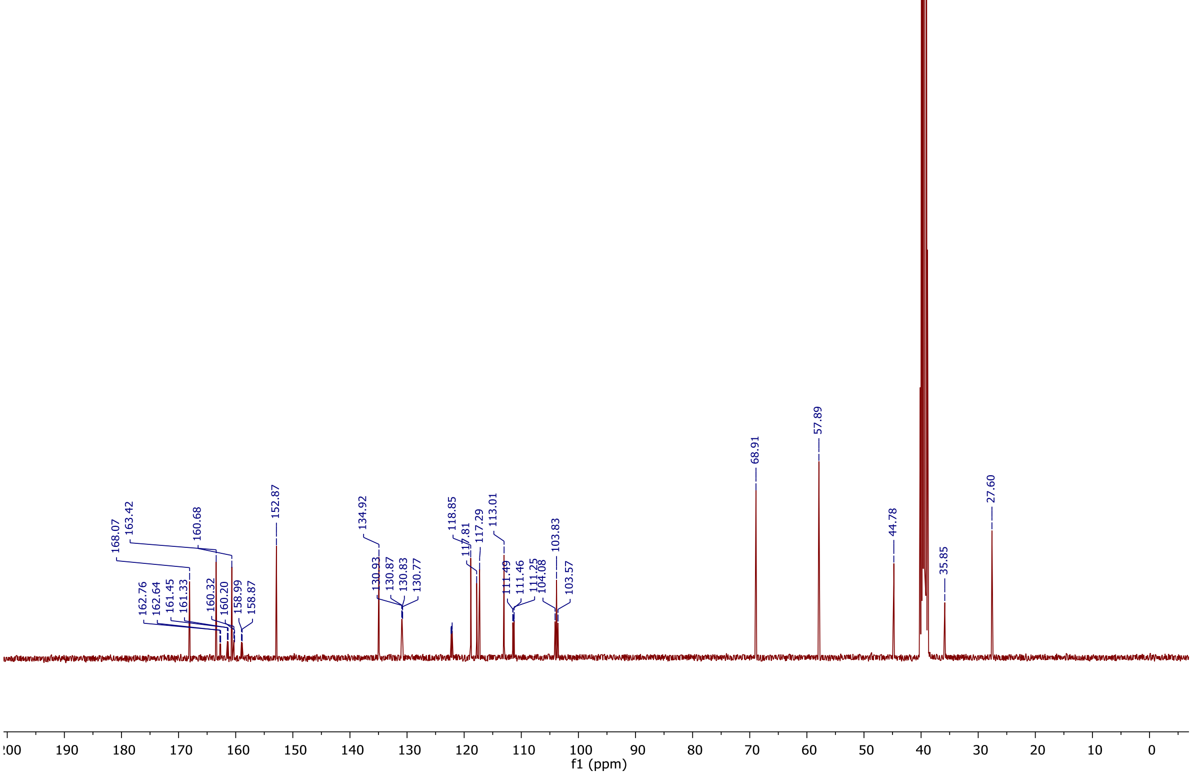

^{1}H NMR (400 MHz, DMSO-d_6)

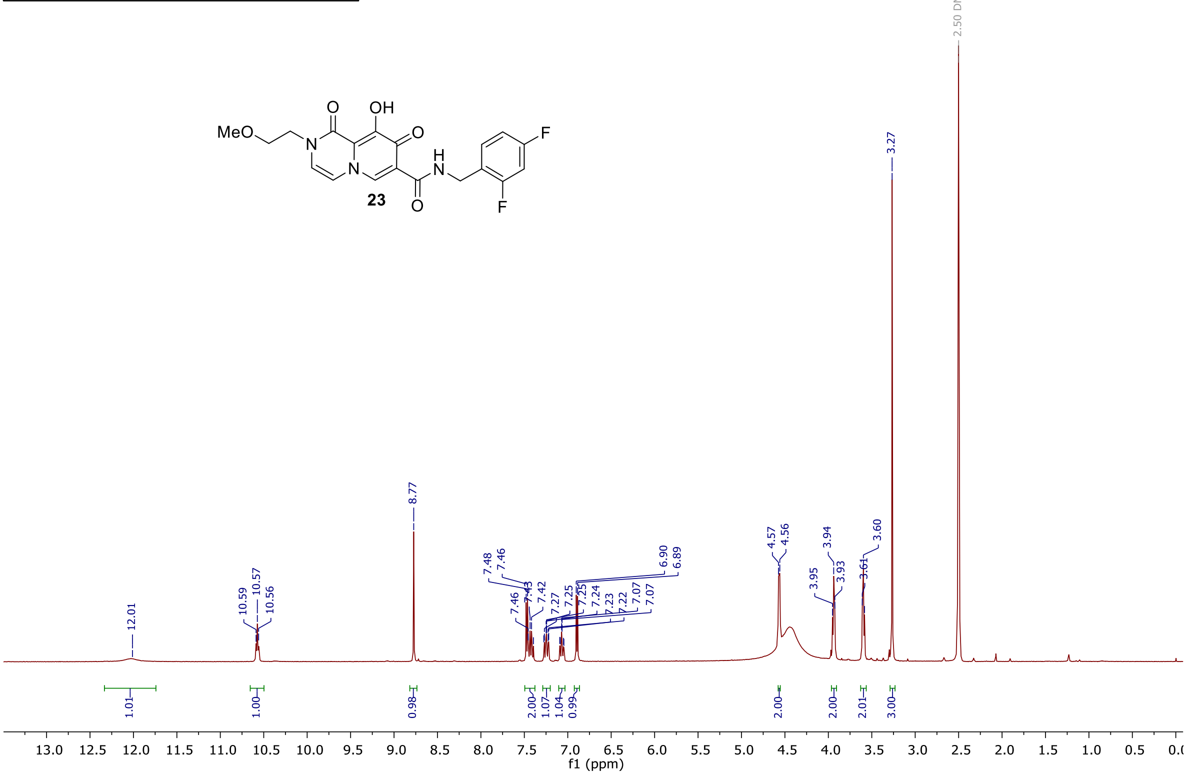

^{13}C NMR (101 MHz, DMSO-d_6)

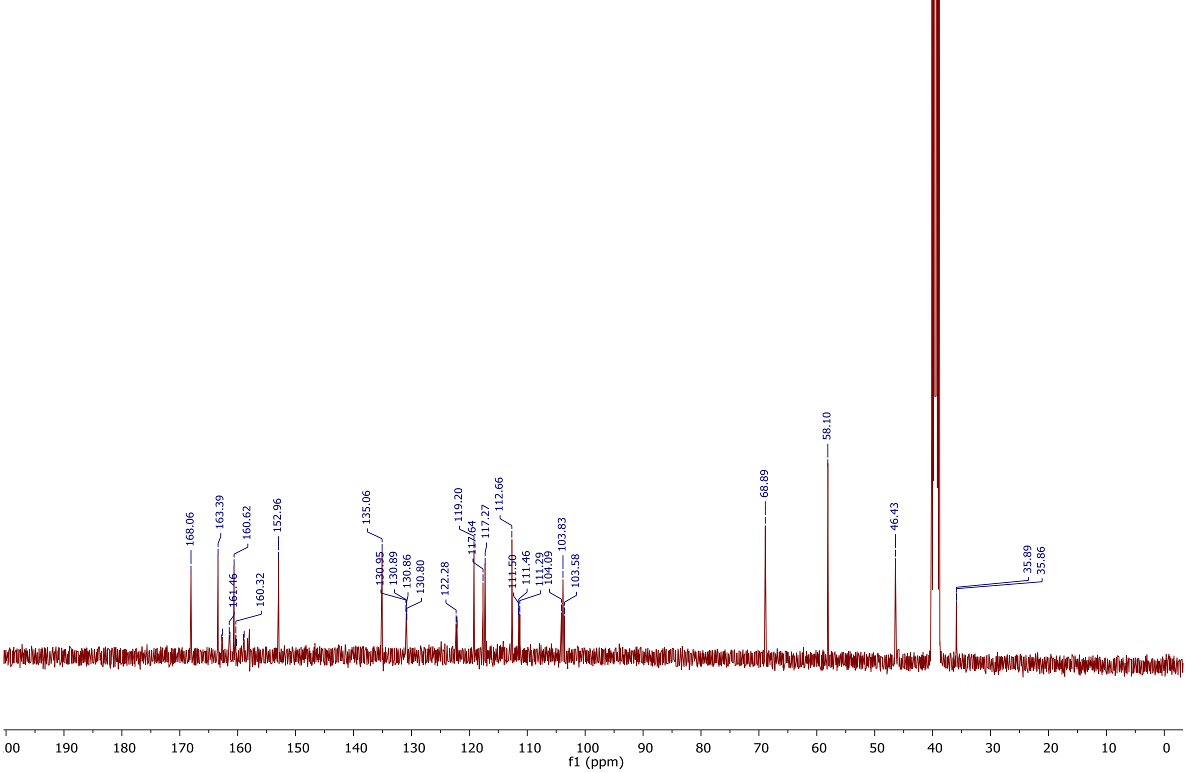

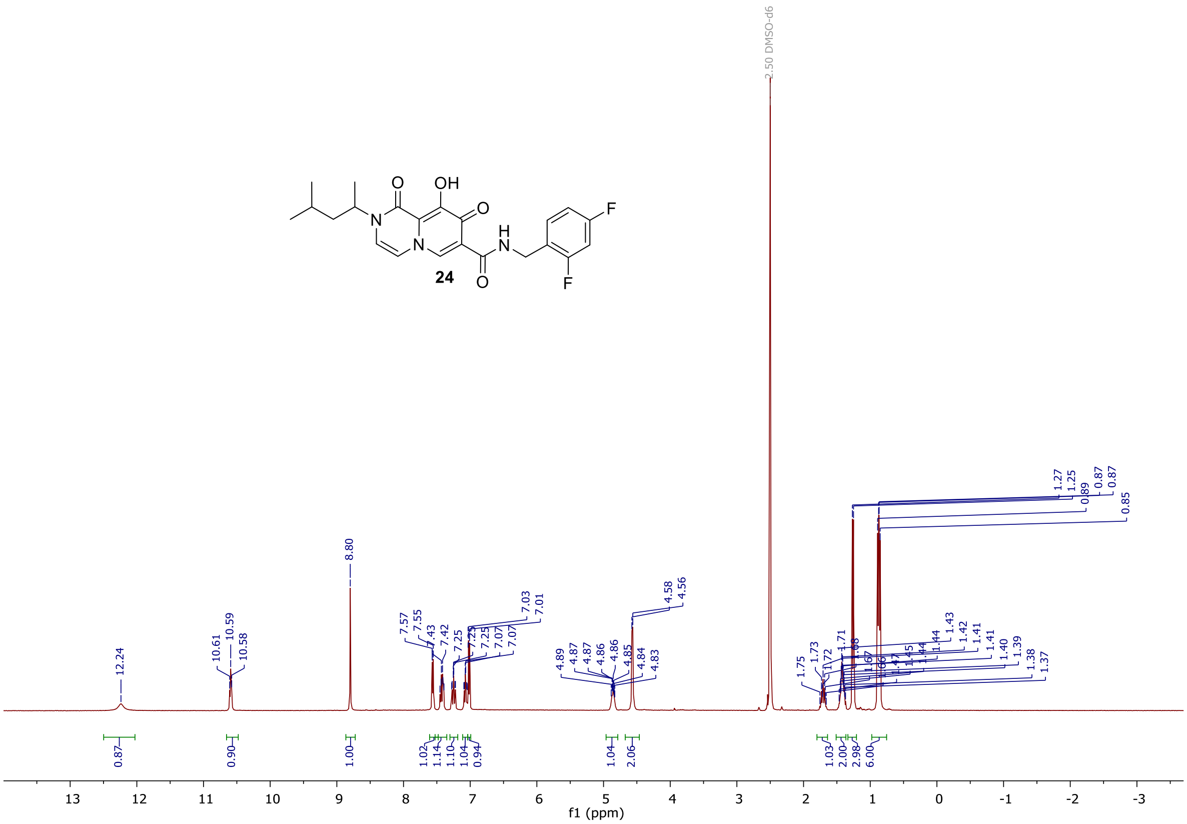
OH
O
N
N
H
F
F
O
O
24
2.50 DMSO-d6
f1 (ppm)

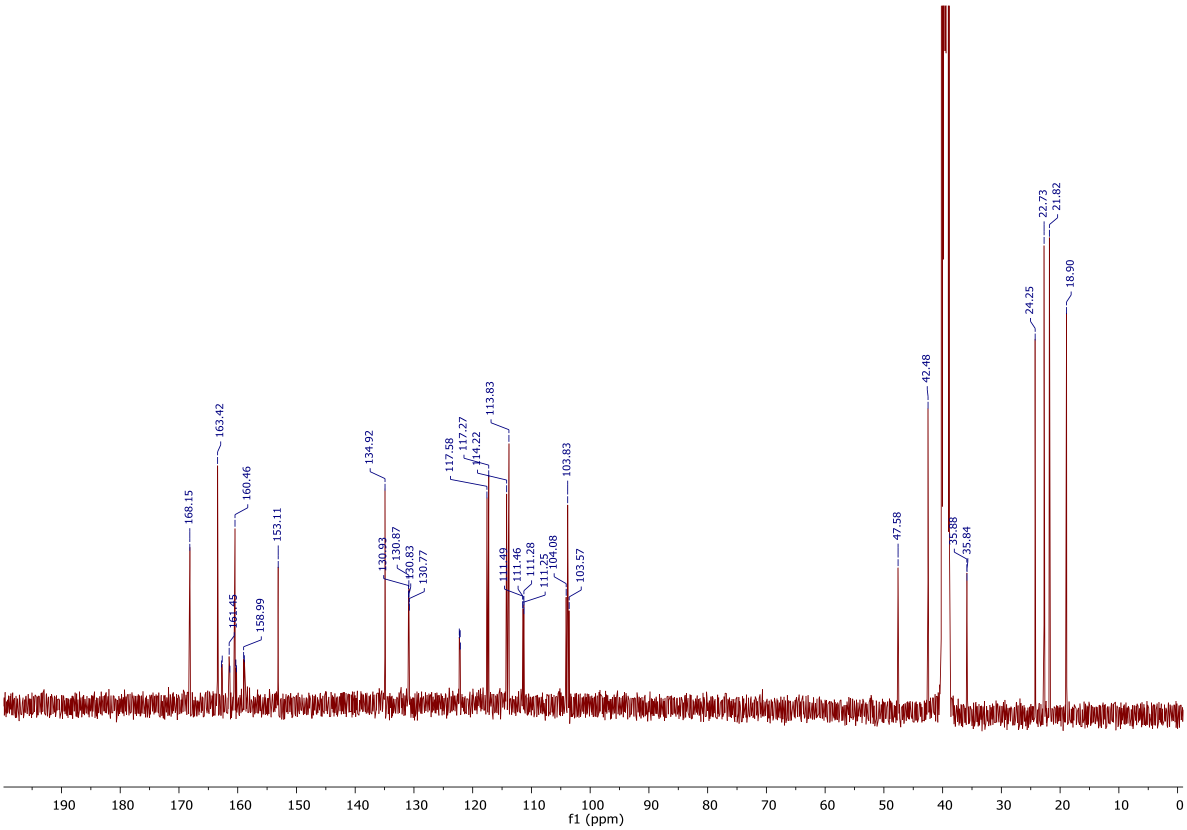
f1 (ppm)

^{1}H NMR (400 MHz, DMSO-d_6)

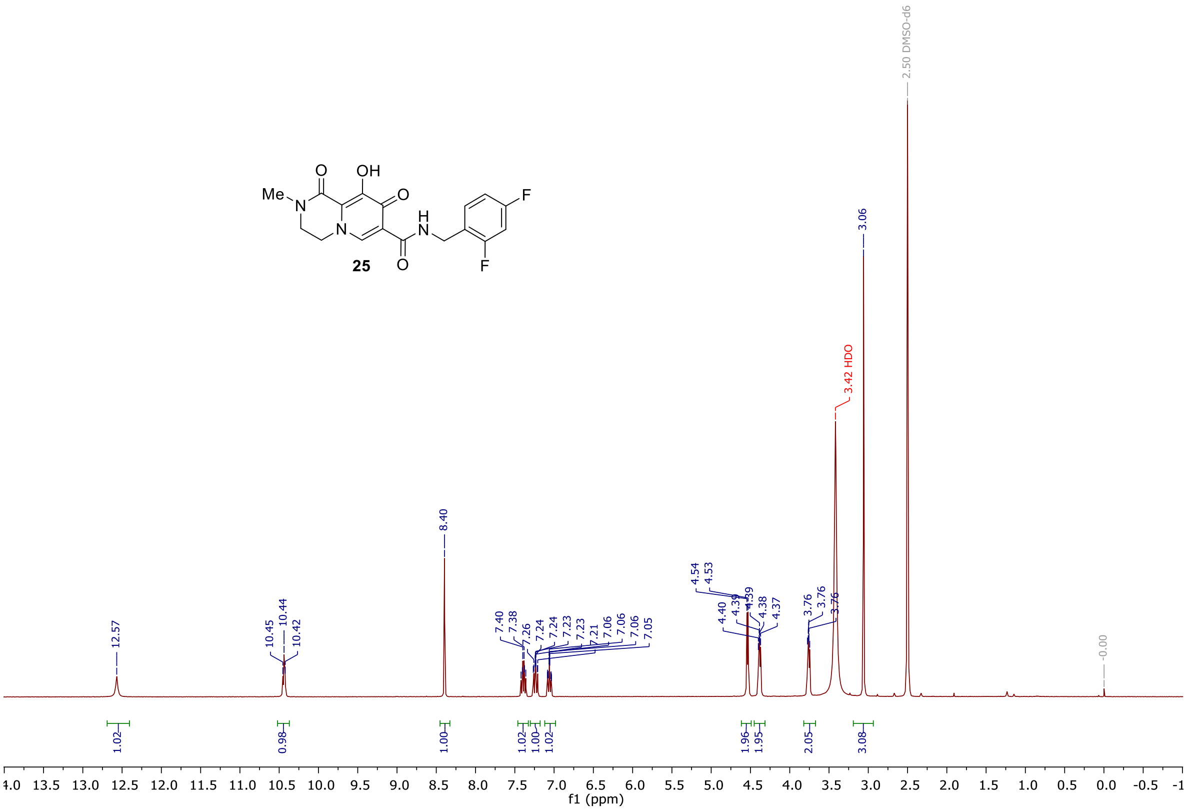

^{13}C NMR (101 MHz, DMSO-d_6)

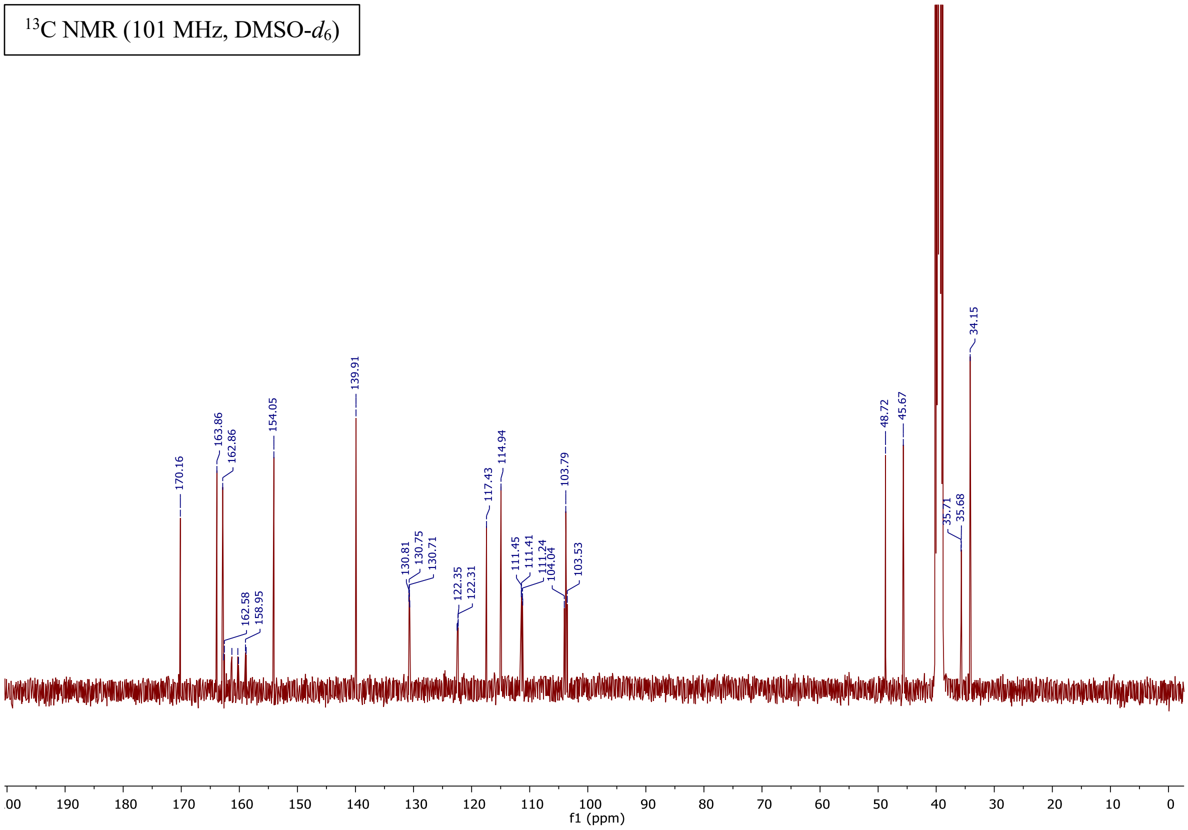

^{1}H NMR (400 MHz, DMSO-d_6)

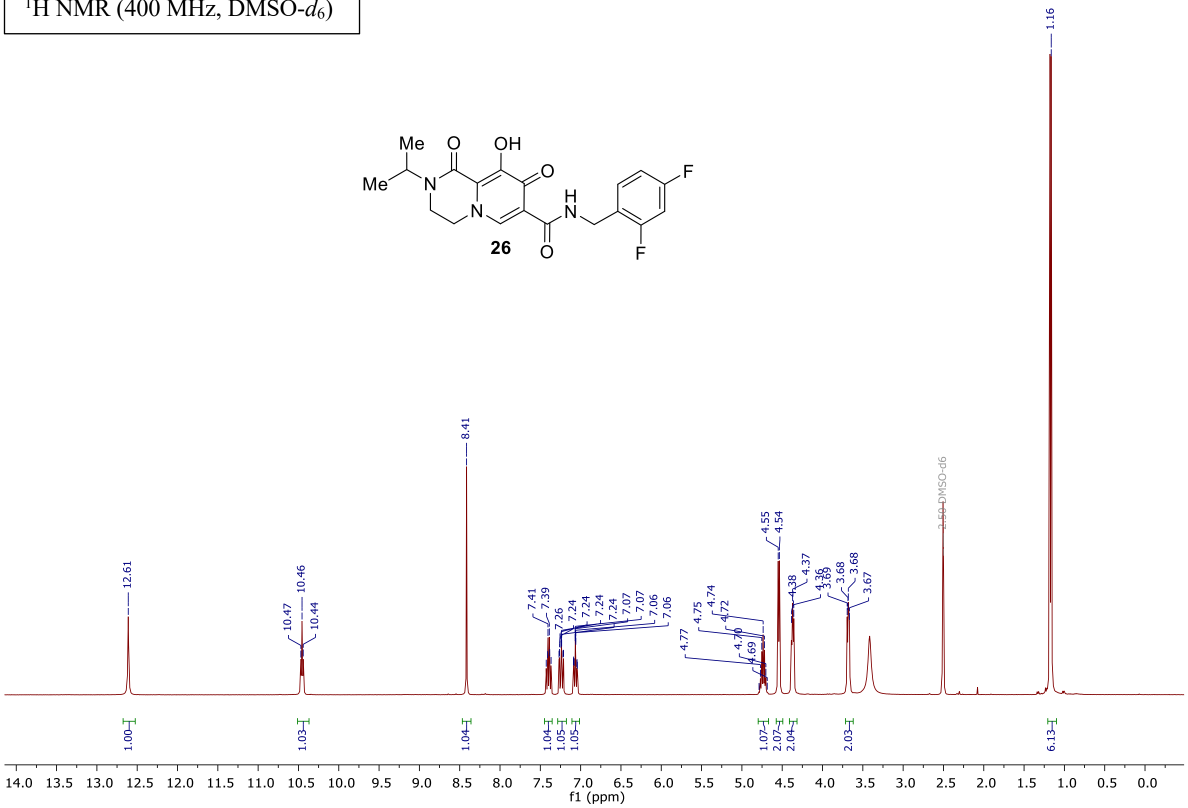

^{13}C NMR (101 MHz, DMSO-d_6)

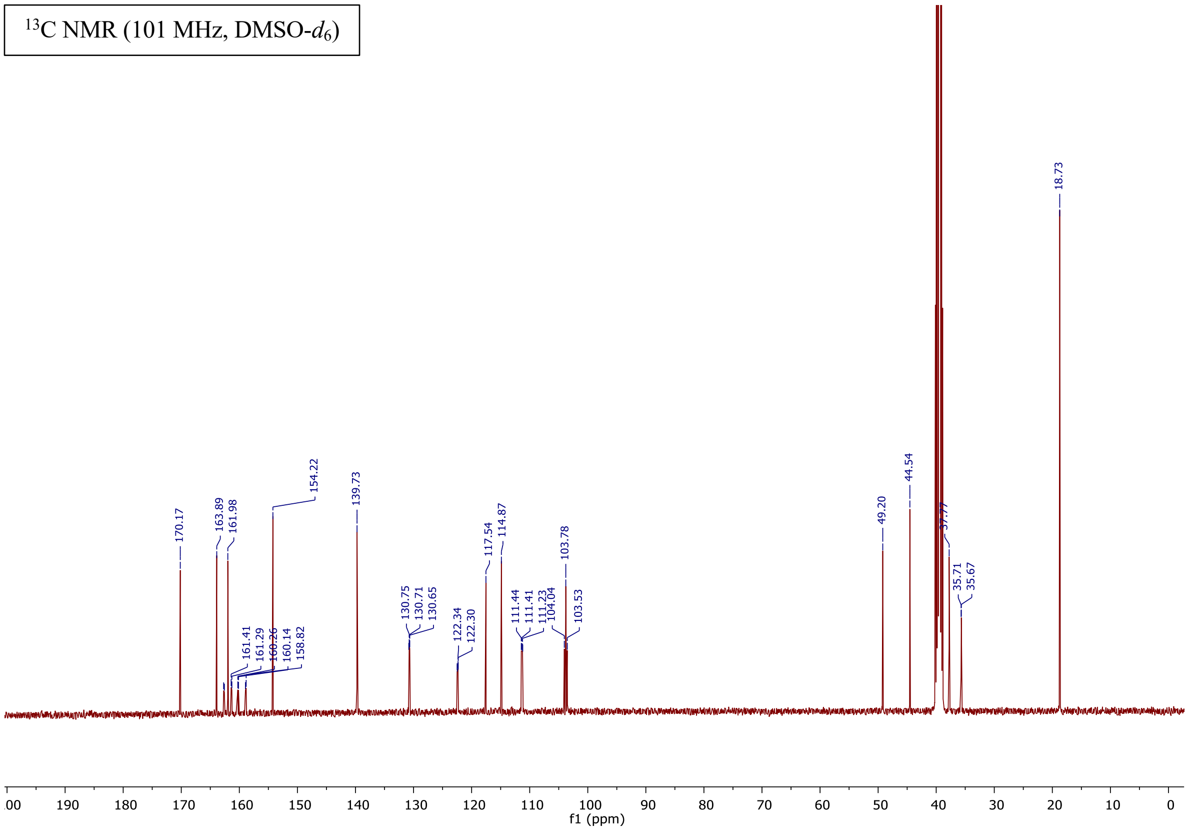

^{1}H NMR (400 MHz, DMSO-d_6)

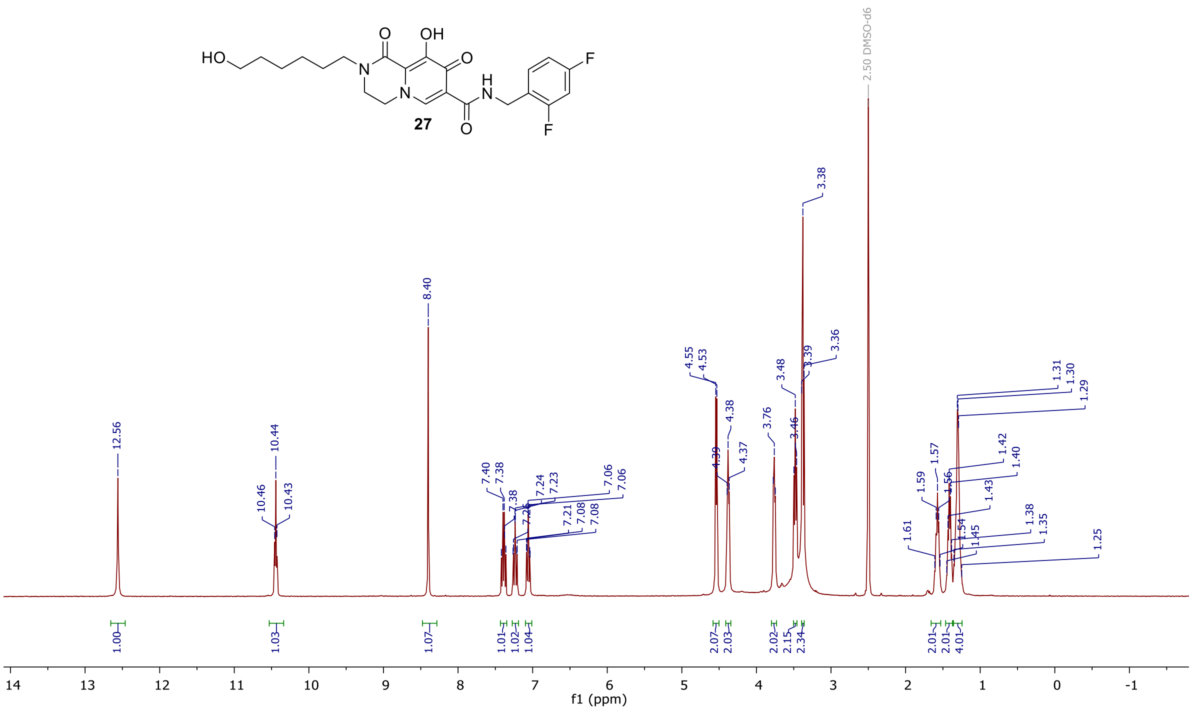

^{13}C NMR (101 MHz, DMSO-d_6)

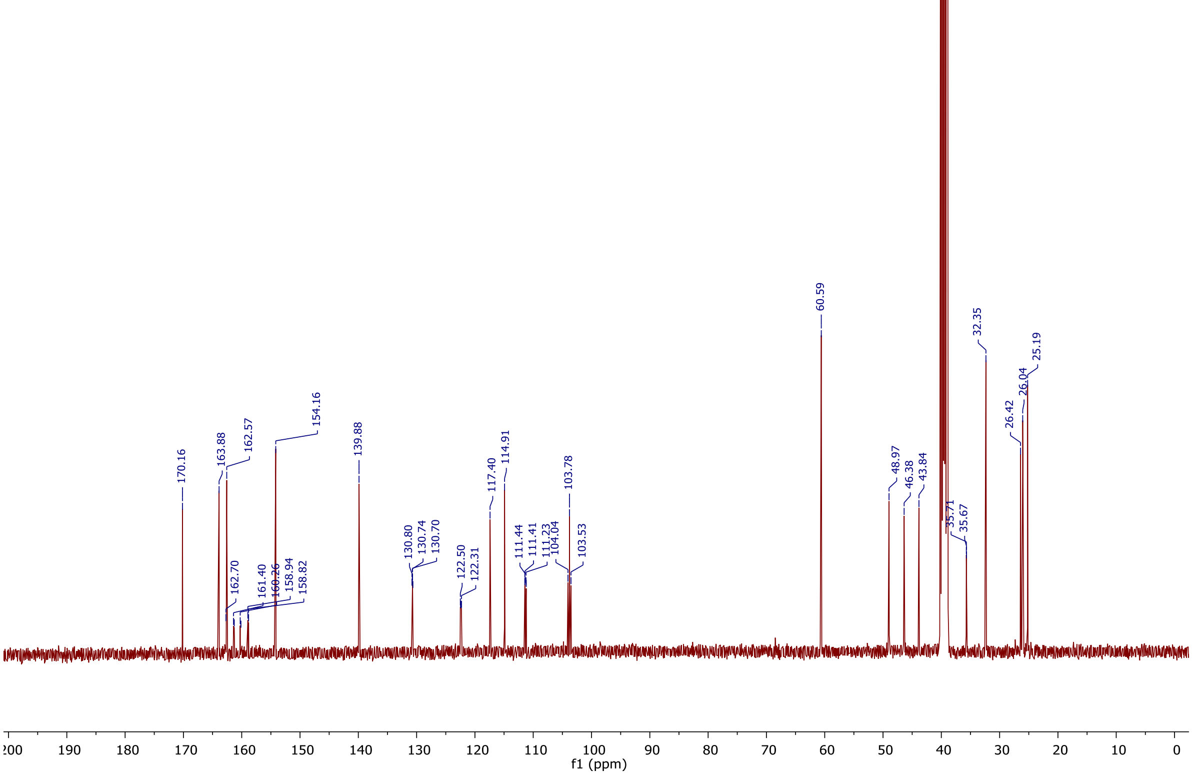

^{1}H NMR (400 MHz, DMSO-d_6)

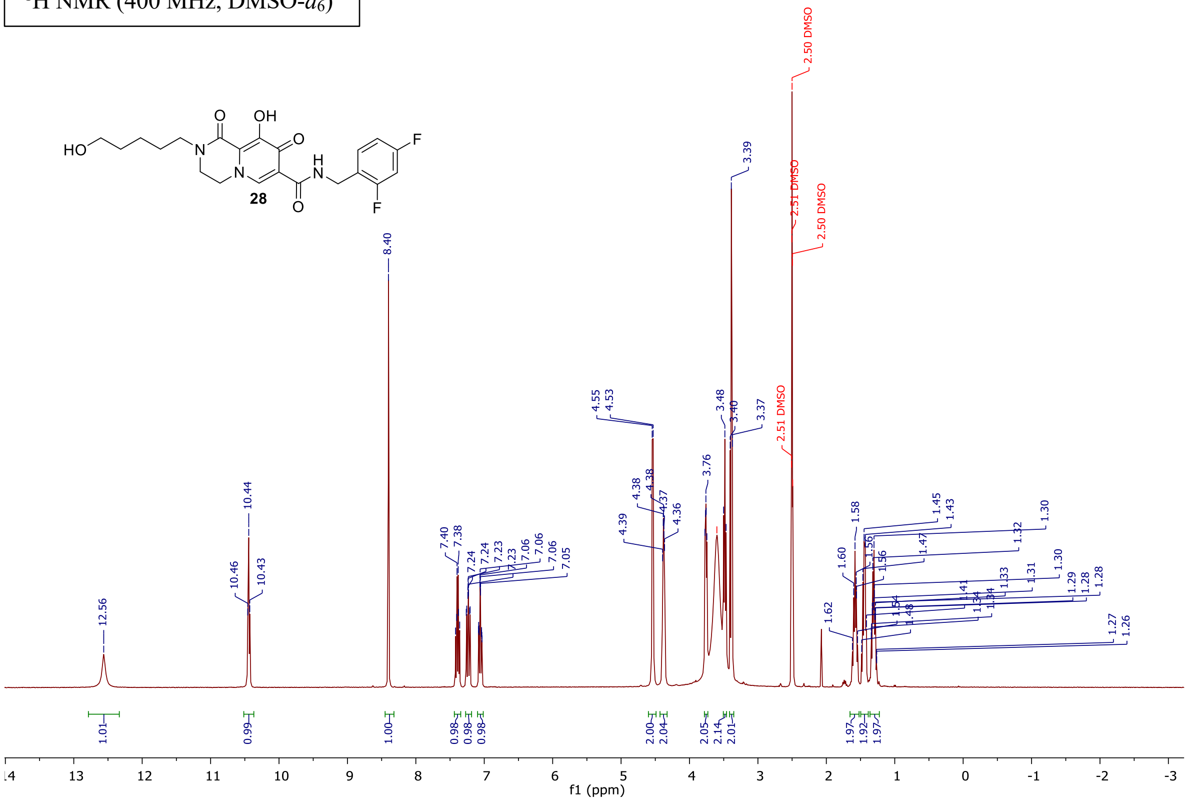

^{13}C NMR (101 MHz, DMSO-d_6)

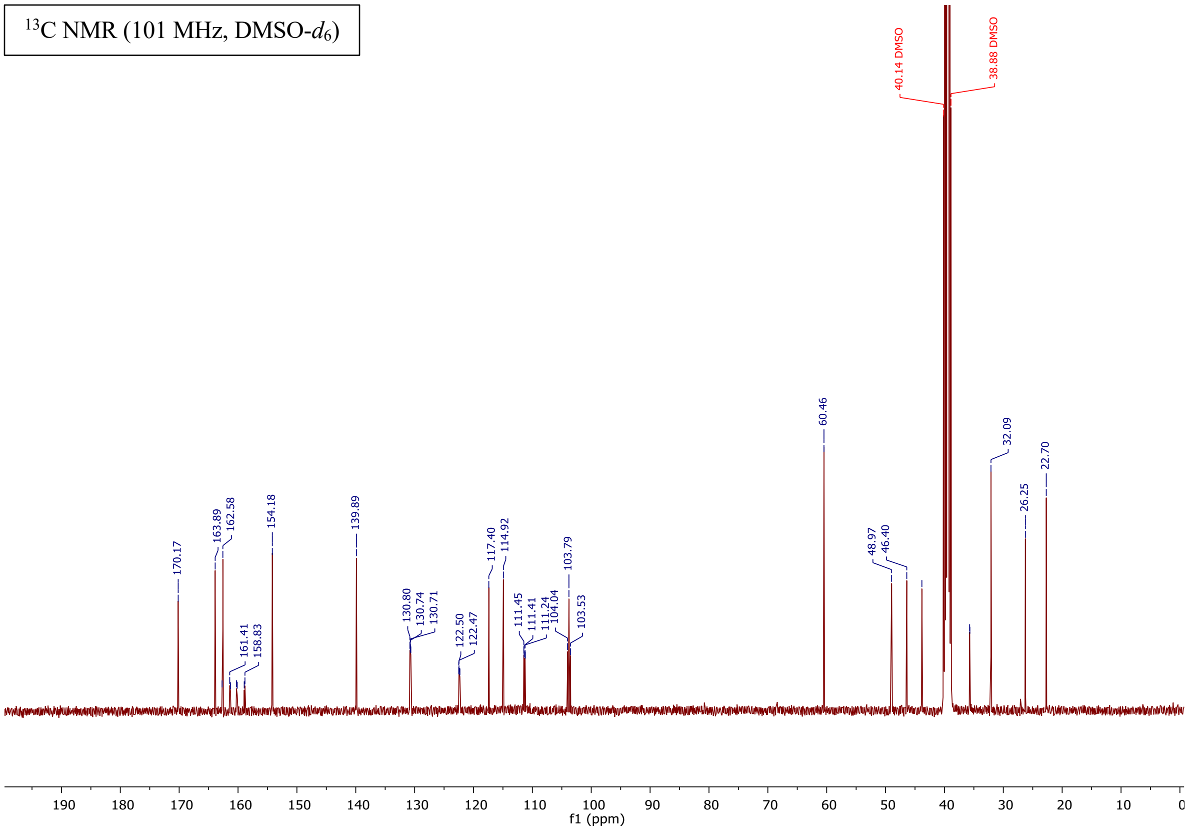

^{1}H NMR (400 MHz, DMSO-d_6)

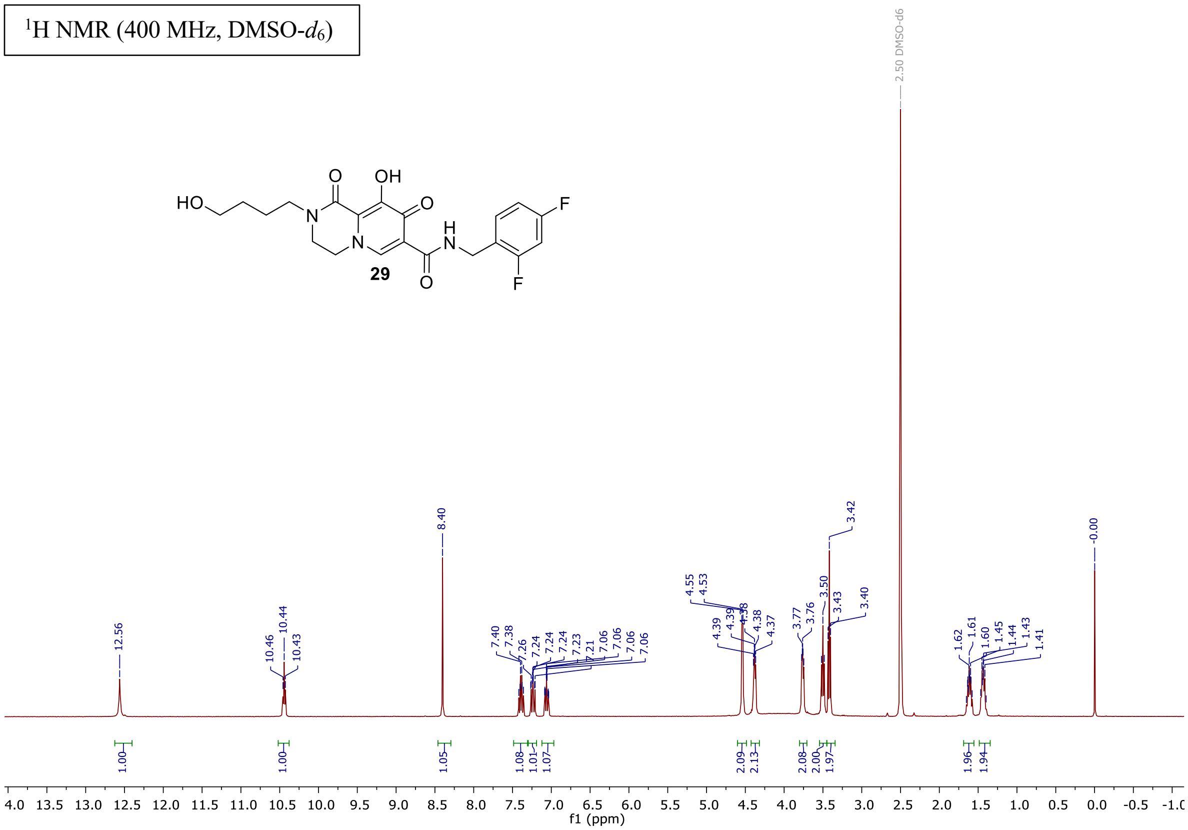

^{13}C NMR (101 MHz, DMSO-d_6)

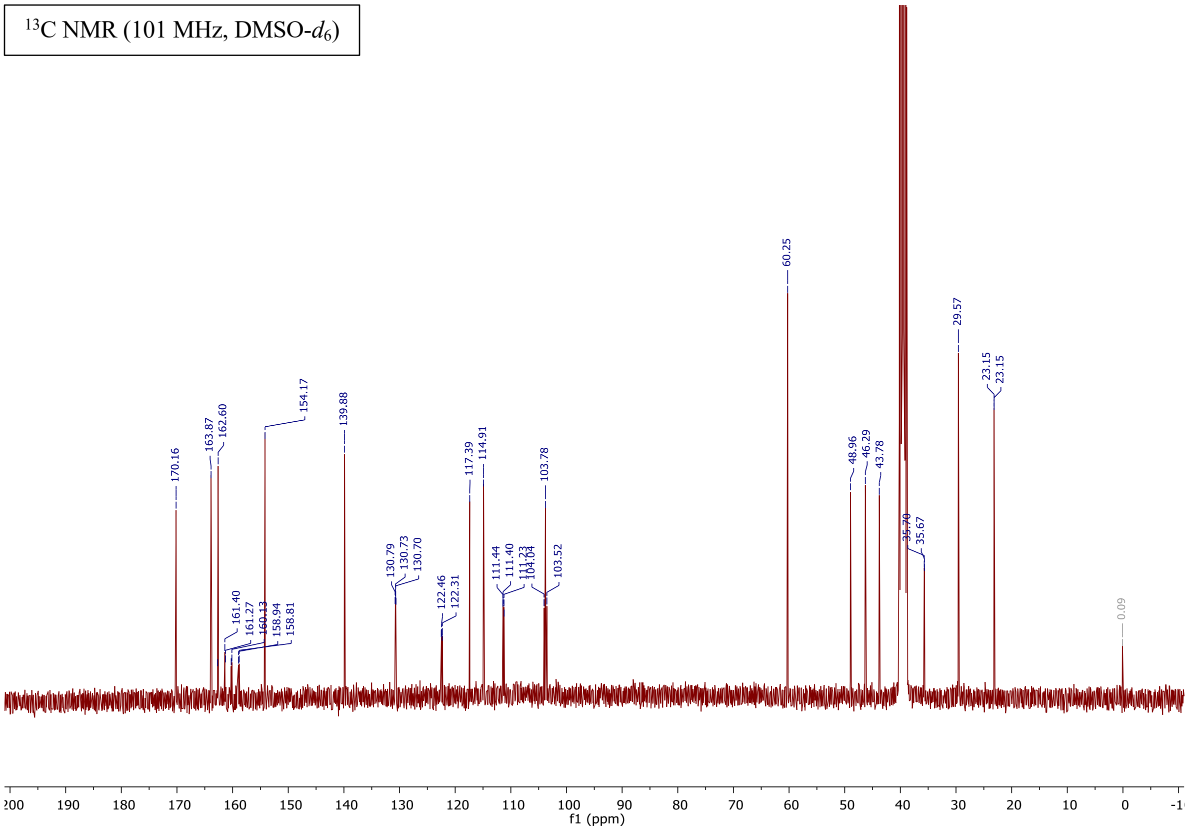

^{1}H NMR (400 MHz, DMSO-d_6)

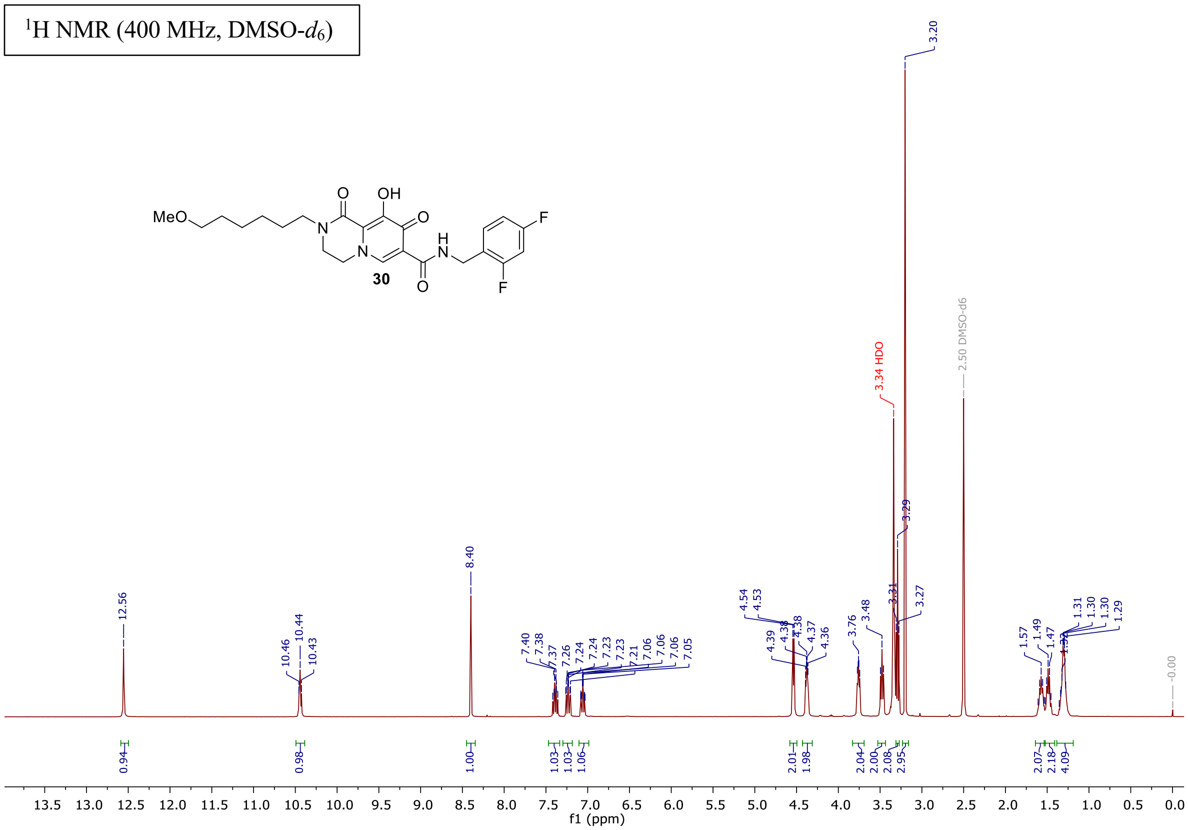

^{13}C NMR (101 MHz, DMSO-d_6)

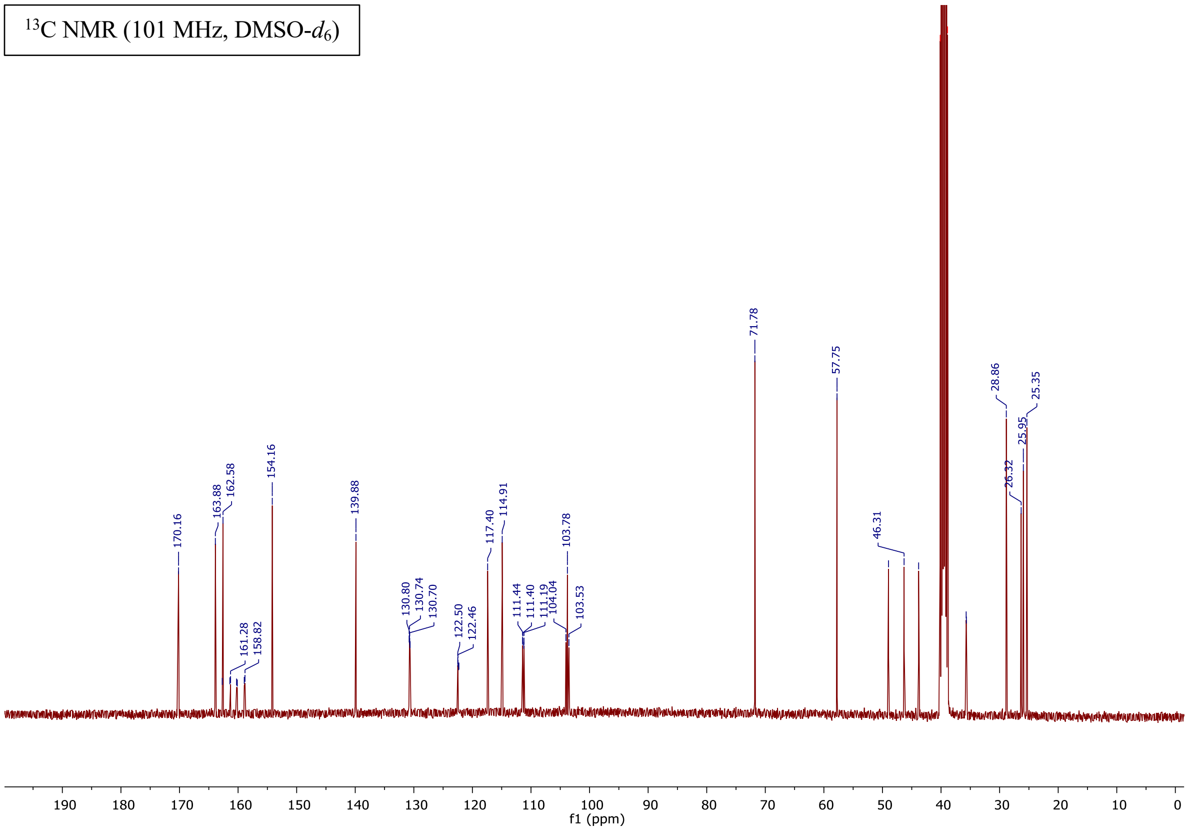

^{1}H NMR (400 MHz, DMSO-d_6)

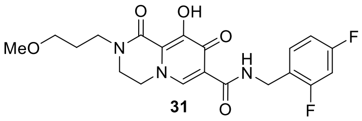

31

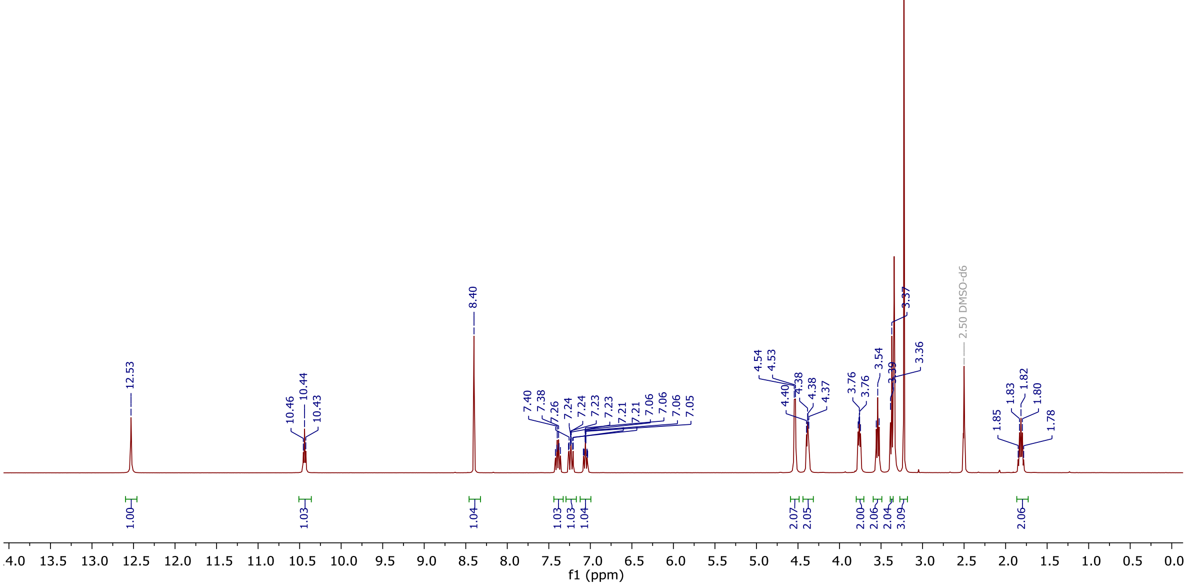

^{13}C NMR (101 MHz, DMSO-d_6)

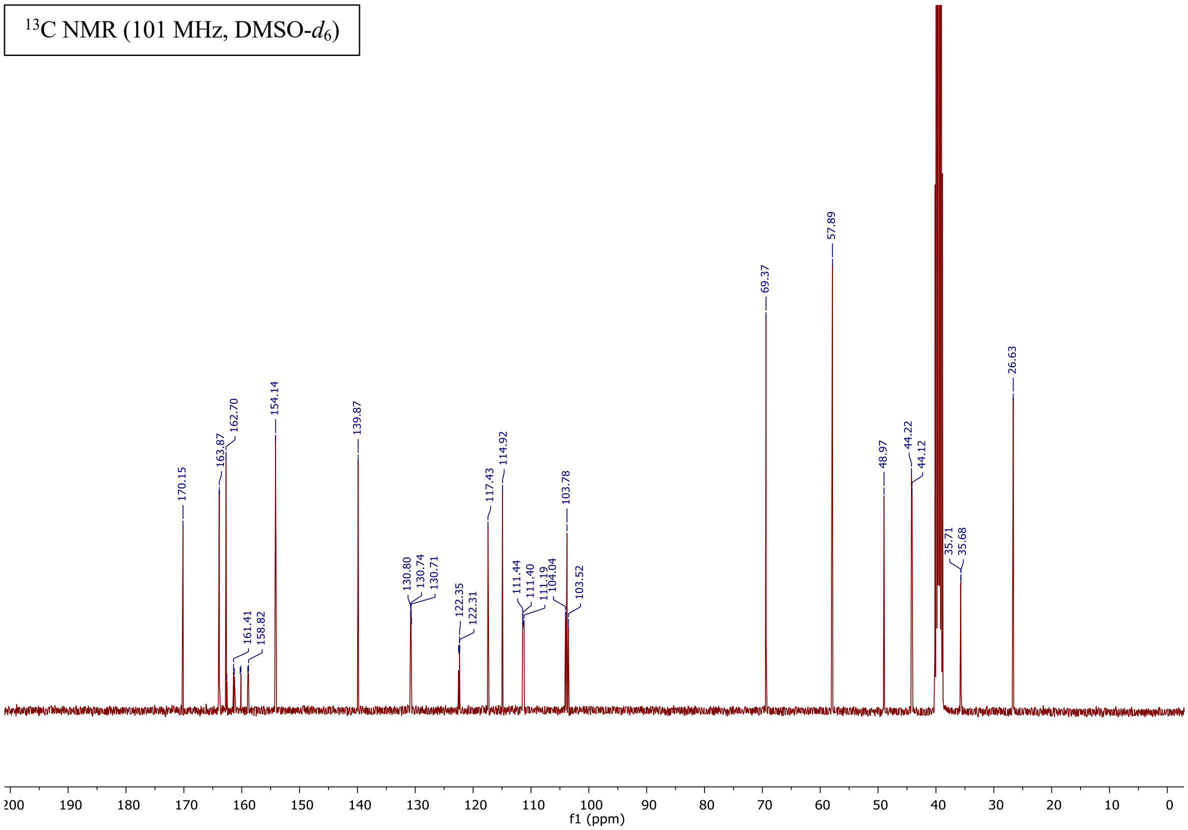

^{1}H NMR (400 MHz, DMSO-d_6)

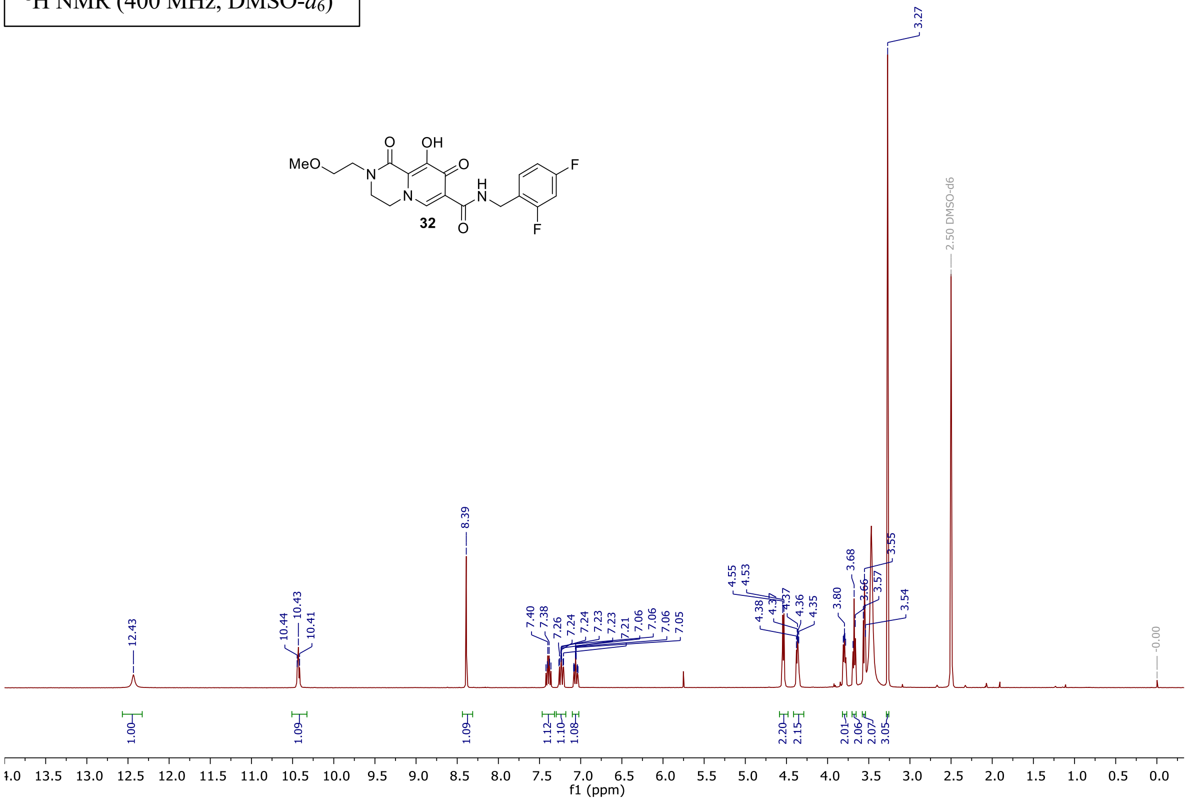

^{13}C NMR (101 MHz, DMSO-d_6)

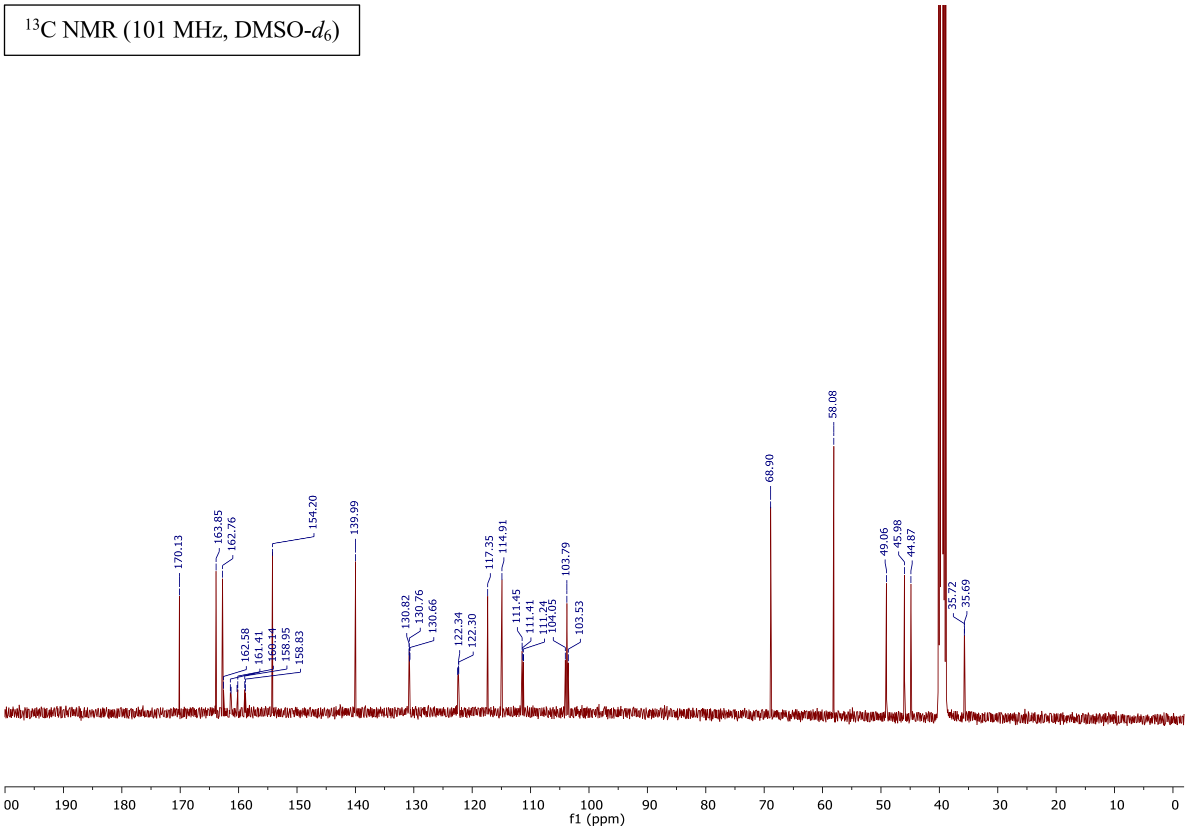

^{1}H NMR (400 MHz, DMSO-d_6)

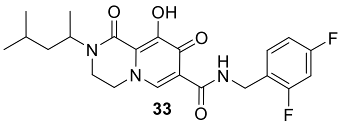

33

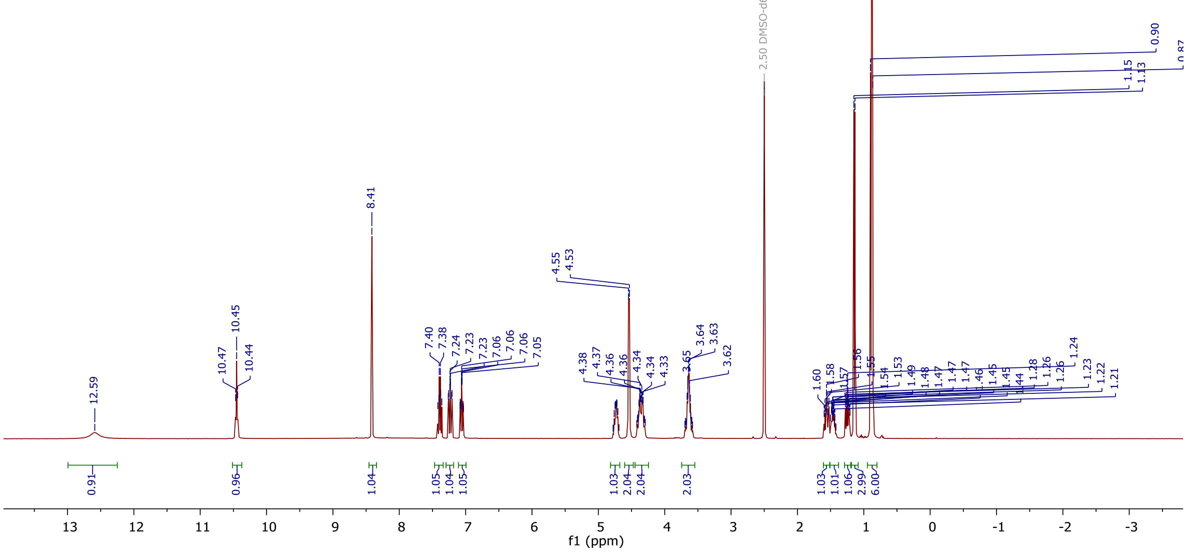

^{13}C NMR (101 MHz, DMSO-d_6)

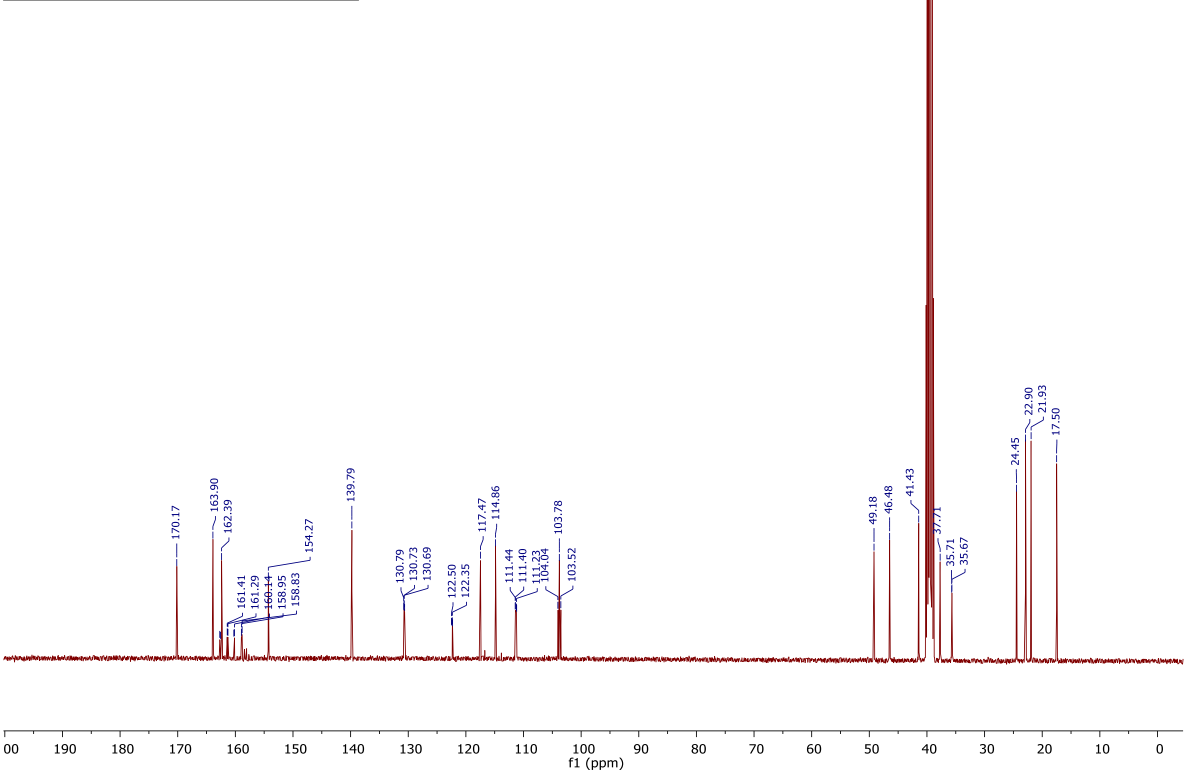

Supplement: Supplementary file 1 [file molecules-28-01428-s001.zip › molecules-2082188-supplementary.pdf]
